# Supplementary material for: Evaluation of Health in Pregnancy grants in Scotland: a protocol for a natural experiment
Source: BMJ Open. 2014 Oct 16;4(10):e006547. doi: 10.1136/bmjopen-2014-006547 (PMC4202003; doi:10.1136/bmjopen-2014-006547)
Supplement: Author's manuscript [file bmjopen-2014-006547.draft_revisions.pdf]

BMJ Open

Evaluation of Health in Pregnancy grants in Scotland: a natural experiment

Journal:	*BMJ Open*
Manuscript ID:	bmjopen-2014-006547
Article Type:	Protocol
Date Submitted by the Author:	04-Sep-2014
Complete List of Authors:	Dundas, Ruth; University of Glasgow, MRC/CSO Social and Public Health Sciences Unit<br>Ouédraogo, Samiratou; University of Glasgow, MRC/CSO Social and Public Health Sciences Unit<br>Bond, Lyndal; Centre of Excellence in Intervention and Prevention Science,<br>Briggs, Andrew; University of Glasgow, Health Economics & Health Technology Assessment Institute of Health & Wellbeing<br>Chalmers, James; NHS National Services Scotland, Information Services Division<br>Gray, Ron; University of Oxford, National Perinatal Epidemiology Unit<br>Wood, Rachael; NHS National Services Scotland, Information Services Division<br>Leyland, Alastair; University of Glasgow, MRC/CSO Social and Public Health Sciences Unit
**Primary Subject Heading**:	Health policy
Secondary Subject Heading:	Epidemiology, Public health, Health economics, Research methods
Keywords:	EPIDEMIOLOGY, Reproductive medicine < GYNAECOLOGY, HEALTH ECONOMICS, Health policy < HEALTH SERVICES ADMINISTRATION & MANAGEMENT, NEONATOLOGY, Maternal medicine < OBSTETRICS

SCHOLARONE™
Manuscripts

PROJECT PROTOCOL

Title: Evaluation of Health in Pregnancy grants in Scotland: a natural experiment

Authors: Ruth Dundas[1], Samiratou Ouédraogo[1], Lyndal Bond[2], Andrew H Briggs[3], James Chalmers[4], Ron Gray[5], Rachael Wood[4], Alastair H Leyland[1]

Affiliations:

[1] MRC/CSO Social and Public Health Sciences Unit, University of Glasgow, Glasgow, UK
[2] Centre of Excellence in Intervention and Prevention Science, Melbourne, Australia
[3] Health Economics and Health Technology Assessment, University of Glasgow, Glasgow, UK
[4] Information Services Division, NHS National Services Scotland, Edinburgh, UK
[5] National Perinatal Epidemiology Unit, University of Oxford, UK

Correspondence to:

Ruth Dundas

MRC/CSO Social and Public Health Sciences Unit, University of Glasgow
200 Renfield Street, Glasgow G2 3QB, Scotland
Tel: +44 141 353 7541 Email: ruth.dundas@glasgow.ac.uk

Keywords: Low birthweight; interrupted time series; multilevel analysis; Health in Pregnancy grant; natural experiment.

For peer review only - http://bmjopen.bmj.com/site/about/guidelines.xhtml

ABSTRACT

Introduction

A substantial proportion of low birthweight (LBW) is attributable to the mother's cultural and socioeconomic circumstances. Early childhood programmes have been widely developed to improve child outcomes. In the UK, the Health in Pregnancy (HiP) grant, a universal conditional cash transfer of £190, was introduced for women reaching the 25th week of pregnancy with a due date on/or after 6th April 2009 and subsequently withdrawn for women reaching the 25th week of pregnancy on/or after 1st January 2011. The current study focuses on the evaluation of the effectiveness and cost-effectiveness of the HiP grant.

Methods and analysis

The population under study will be all singleton births in Scotland over the periods of January 2004 to March 2009 (pre-intervention), April 2009 to April 2011 (intervention) and May 2011 to December 2013 (post-intervention). Data will be extracted from the Scottish maternity and neonatal database. The analysis period 2004-2013 should yield over 585,000 births. The primary outcome will be birthweight among singleton births. Other secondary outcomes will include gestation at booking, booking before 25 weeks; measures of size and stage; gestational age at delivery; weight-for-dates, term at birth; birth outcomes and maternal smoking. The main statistical method we will use is interrupted time series. Outcomes will be measured on individual births nested within mothers, with mothers themselves clustered within datazones. Multilevel regression models will be used to determine whether the outcomes changed during the period in which the HiP grants was in effect. Subgroup analyses will be conducted for those groups most likely to benefit from the payments.

Ethics and dissemination

Approval for data collection, storage and release for research purpose has been given (6th of May 2014, PAC38A/13) by the Privacy Advisory Committee. The results of this study will be disseminated through peer-reviewed publications in journals, national and international conferences.

For peer review only - http://bmjopen.bmj.com/site/about/guidelines.xhtml

ARTICLE SUMMARY

Article focus

- To evaluate the effectiveness and cost-effectiveness of Health in Pregnancy grant (HiP) in Scotland.

Key messages

- We will assess the difference in birthweight for babies born to those mothers who were eligible for the HiP grant with babies born before the HiP grant was introduced or after it was withdrawn using interrupted time series multilevel analysis.
- Subgroup analyses will be conducted for those groups seen as having the greatest potential to benefit from the payments such as those living in the most deprived areas, those in the lowest social classes, lone mothers, primiparous women, teen mothers, mothers from ethnic minorities and selected combinations of these groups.
- As part of the project an economic model will be developed based on a review of the literature to relate birthweight changes to long-term cost and health outcomes (in terms of QALYs).

Strengths and limitations of this study

The strengths of this study are:

- This is the first study evaluating the effect and the cost-effectiveness of the HiP grant in Scotland. It will use routinely available vital event and maternal and neonatal health records that are known to have high completeness and accuracy.
- The evaluation of the HiP grant using an interrupted time series design will enable us to analyse birthweight trends in Scotland and detect whether the intervention has had an effect over and above the underlying temporal trend. The use of interrupted time series will overcome other biases such as the autocorrelation of repeated measurements (measurement taken close together are related), seasonal effects (birthweight varies according to month of birth), the duration of the intervention (we will have pre-intervention intervention and post-intervention), and random variation in the measurement (birthweight).

The limitations of this study

For peer review only

For peer review only - http://bmjopen.bmj.com/site/about/guidelines.xhtml

- This study will evaluate the effect and the cost-effectiveness of the HiP grant, birthweight and other birth outcomes in Scotland using obstetric as well as maternal data. However, these outcomes may be influenced by many factors, not all of which are routinely captured (such as maternal diet, maternal work and psychological stress, abuse, exposure to toxic substance).[11]
- The HiP grant was money given to pregnant women with no constraint on its use. The use of routine data gives us no indication on how the money was spent.

For peer review only

For peer review only - http://bmjopen.bmj.com/site/about/guidelines.xhtml

INTRODUCTION

Low birthweight (LBW) due to preterm birth, intrauterine growth restriction, or being born small for gestational age (SGA), is commonly associated with perinatal mortality and impaired development[1-11]. Despite the improvement of the mortality in LBW babies over the past three decades, more than 70% of all neonatal mortality in Europe is found in infants weighing less than 2500 grams (g).[12]

LBW refers to birth weights below 2500 g,[13] irrespective of the gestational age of the infant. In most developed countries, the prevalence of LBW has increased due to several reasons: the number of multiple births with the increased risks of pre-term births and low birth weight partly as a result of the rise in fertility treatments; older age at childbearing and increases in the use of delivery management techniques such as induction of labour and caesarean delivery, which have increased the survival rates of low birth weight babies.[14] About one in 20 babies born in Europe in 2010 weighed less than 2500 g at birth.[12] With LBW estimated at 7.0% of live births in England and Wales, 6.5% in Scotland and 5.7% of live births in Northern Ireland, prevalence of LBW in the countries of the United Kingdom (UK) tended to be higher than in the rest of Europe.[12 15] Moreover, compared with other Western European countries, the UK has an incidence of LBW (<2500g) and very LBW (<1500g) in the top third. The proportion of pre-term birth (<37 weeks) is also high compared with other Western European countries.

Considerable attention has been focused on the causal determinants of LBW, in order to identify potentially modifiable factors. A substantial proportion of LBW is attributable to the mother's cultural and socioeconomic circumstances such as socioeconomic status (SES), harmful behaviours (smoking and excessive alcohol consumption) and poor nutrition during pregnancy.[11 16-18] In a study of social class inequalities in perinatal outcomes in Scotland, Fairley and Leyland[19] reported a percentage of 5.8% LBW in unskilled social class (V) compared to 2.9% in professional social class (I) between 1995 and 2000. The systematic review and meta-analyses of social inequality and infant health in the UK performed by Weightman et al.[20] found that the odds ratio for low birth weight was 1.79 (95% CI 1.43 to 2.24) in the lowest compared to the highest social class. This effect may vary with maternal factors such as age and smoking status. Smoking during pregnancy reduces birthweight by 162 to 377 g, depending on daily consumption (larger reduction for heavy smokers) and the trimester in which exposure occurs (larger reduction during the last trimester).[18 21-22]

For peer review only - http://bmjopen.bmj.com/site/about/guidelines.xhtml

The association between maternal nutrition and birth outcome is complex and is influenced by many biologic, socioeconomic, and demographic factors, which vary widely in different populations.[23-24] However, it has been reported that favourable prenatal nutrition associated with adequate prenatal care can have a positive impact on birth outcomes and morbidity in adult life.[25-26] Indeed, the developmental model of the origins of chronic disease proposes the causal influence of undernutrition *in utero* on coronary heart disease and stroke in adult life.[7-9] An improvement in fetal nutrition may therefore have far reaching consequences in terms of the prevention of disease. A review of maternal nutrition and birth outcomes identified improving maternal nutrition as being beneficial to the prevention of adverse birth outcomes in lower social class groups.[24]

A number of early childhood programmes have been developed to improve child outcomes. There is mixed evidence that these programmes do provide such improvement. In a meta-analysis of the effect of interventions in pregnancy on maternal and obstetric outcomes, Thangaratinam et al. concluded that dietary and lifestyle interventions in pregnancy reduced maternal gestational weight gain but had less effect on outcomes related to fetal weight and other morbidity and mortality.[27] Glassman et al.[28] who reported the results of conditional cash transfer programmes increasingly being adopted and scaled in developing countries, found that the programmes have increased the uptake of maternal and newborn health services, especially skilled attendance at delivery and antenatal monitoring. However, the impact of the programs on maternal and newborn mortality has not been well documented. Therefore, they recommended more rigorous impact evaluations that document impact pathways and take factors, such as cost-effectiveness, into account. Other studies evaluating payments to influence health behaviour found financial incentives were effective in increasing infrequent behaviours such as attending clinic appointments particularly in low-income groups, and recommended payments as being more effective than information and less restrictive than legislation.[29-31] In Canada, Brownell et al. have evaluated a complex programme on a prenatal benefit provided to families on low income during pregnancy.[32] They found that the receipt of this prenatal benefit was associated with a reduction in incidence of both low birthweight babies and preterm births. They suggested that efforts should be made to ensure all low-income women receive the income supplement.

In the UK, the Health in Pregnancy (HiP) grant was introduced for women reaching the 25th week of pregnancy with a due date on/or after 6th April 2009. It was subsequently withdrawn for women reaching the 25th week of pregnancy on/or after 1st January 2011. The HiP grant

For peer review only

was a universal conditional cash transfer of £190 for women reaching 25 weeks of pregnancy if they had sought health advice from a doctor or midwife. It was designed to provide additional financial support, in the last months of pregnancy, towards a healthy lifestyle including diet, and it was suggested that the link to the requirement for pregnant women to seek health advice from a professional may provide a greater incentive for expectant mothers to seek the recommended health advice at the appropriate time. The grant was paid and administered by Her Majesty's Revenue and Customs (HMRC) on receipt of a claim form partly completed by the midwife or doctor. Advice was offered as normal by doctors and midwives. Payment was made directly into a bank account with a telephone helpline available to provide support through the claims process including options for payment in the event of difficulties opening a bank account. Take up of the grant was said to be about the same level as for child benefit (98-99%, personal communication, HMRC).

The current study focuses on the evaluation of the effectiveness and cost-effectiveness of the HiP grant. As a primary outcome, we will consider the difference in birthweight for babies born to those mothers who were eligible for the HiP grant with babies born before the HiP grant was introduced or after it was withdrawn. Specific questions the research project will address are:

- Were there differential impacts of the intervention for particular subgroups defined by socioeconomic (defined in terms of both area deprivation and individual occupational social class), demographic (marital status, age, maternal height), or obstetric (parity, previous caesarean section) factors or for selected combination of these groups?
- Was the HiP grant cost-effective? How did cost-effectiveness vary across important subgroups identified as having differential outcomes?

The principle of universalism in the allocation of social benefits, that is the availability of social benefits to everyone as of right, is contrasted with allocation on a selective basis (targeted) in which benefits are allocated on the basis of need as determined by means testing of income.[33] The advantage of universal benefits is that they are easy to administer and can be efficiently delivered. The major disadvantage is that they are expensive, because they are delivered to those who do not need them as much as to those who do. However, the use of targeting involves some mechanism that discriminates between the poor and the non-poor. As such it always runs the danger of committing either type I errors which occur when someone

For peer review only - http://bmjopen.bmj.com/site/about/guidelines.xhtml

who deserves the benefits is denied them (underpayment, false positives), or type II errors, which occur when benefits are paid to someone who does not deserve them (overpayment, leakage).[34] The HiP grant represented an attempt to influence behaviour – appropriate and timely receipt of antenatal care advice – by means of a relatively modest, universally applied cash transfer. The evaluation of the effectiveness of such a payment may inform other policies aiming to change behaviour.

For peer review only

For peer review only - http://bmjopen.bmj.com/site/about/guidelines.xhtml

METHODS AND ANALYSIS

Study design

The HiP grant will be evaluated as a natural experiment using interrupted time series analysis to compare outcomes before the introduction of the intervention in Scotland and immediately after its withdrawal with those during the period for which it existed.

The Medical Research Council (MRC) has issued guidelines regarding the use of natural experiments to evaluate population health interventions when exposure to the intervention has not been manipulated by the researchers (Table 1).[35-36]

Table 1: Guidance for use of natural experiments to evaluate population health interventions

When to use a natural experimental approach?	How does the evaluation of HiP* grant meet these criteria?
▪ There is a reasonable expectation that the intervention will have a significant health impact, but scientific uncertainty about the size or nature of the effects.	▪ The HiP grant represented an attempt to influence behaviour – appropriate and timely receipt of antenatal care advice. With the sample size we are using in this evaluation study, we are able to detect small changes in birthweight.
▪ Natural experimental study is the most appropriate method for studying a given type of intervention.	▪ The HiP grant was a universally applied cash transfer available for all pregnant women with no discrimination between socioeconomic classes. This policy was not introduced using a randomised allocation
▪ It is possible to obtain the relevant data from an appropriate study population, comprising groups with different levels of exposure to the intervention.	▪ The uptake of the HiP grant was thought to be 98-99%. The linked Scottish birth dataset has 98% coverage of births and the primary outcome, birthweight, is well measured, 99.9% complete and accurate. Exposure is determined by the dates for which the HiP grant was in existence.
▪ The intervention or the principles behind it have the potential for replication, scalability or generalisability.	▪ The HiP grant is replicable everywhere in countries with similar health systems.

HiP* grant: Health in Pregnancy grant

For peer review only - http://bmjopen.bmj.com/site/about/guidelines.xhtml

The guidance advocates a number of designs including regression discontinuity designs such as interrupted time series. The interrupted time series approach is a powerful tool used for evaluating the impact of a policy change or quality improvement programme on the rate of an outcome in a defined population of individuals.[37-39] This approach will allow us to use a comparison group of pregnant women who delivered before the HiP grant was introduced, an intervention group who received the HiP grant and an additional post-intervention group who delivered after the HiP grant was withdrawn. It will also allow adjustment for seasonality, temporal trends and the socio-demographic and obstetric characteristics of the mother.

Study population

The population under study will be all singleton births in Scotland over the periods of January 2004 to March 2009 (pre-intervention), April 2009 to April 2011 (intervention) and May 2011 to December 2013 (post-intervention).

The Scottish maternity and neonatal database is a comprehensive record linkage system. Probabilistic linkage procedures are used to add a unique identifier to all datasets to ensure all relevant records relating to an individual can be linked as required. It facilitates the linkage of a number of records from the system of Scottish Morbidity Records (SMR) including mother's obstetric records (SMR02) and the baby's birth and neonatal information from Scottish Birth Records (SBR). Further links to the stillbirth and infant Death Survey and the National Records of Scotland (NRS) birth, stillbirth and infant death records can be carried out. The coverage is almost all births in Scotland. From NRS data, we know of all registered births in Scotland (ie 100%). We have suitable record linked data on 98% of these births. These will nearly all be hospital births; a fairly high proportion of the missing records are home births.

There is an average of about 56,000 births per year in Scotland. The analysis period 2004-2013 should yield over 585,000 births. The analysis period 2006-2013 should yield over 455,000 births.

For peer review only - http://bmjopen.bmj.com/site/about/guidelines.xhtml

We have chosen to use data from the Scotland for this evaluation for the following reasons. First, data are available at a national level on the approximately 56,000 deliveries per year. Second, Scotland has a long history of collecting high quality routine data; the coverage, completeness and quality of the data are considered to be very high. Third, the concentration of deprivation within parts of Scotland is unique within the UK. For example six of the ten most deprived electoral constituencies in the UK are in Scotland and using a UK wide Cartstairs index, the Scotland's population is over-represented in the bottom 5 deciles compared to England. Fourth, data on smoking at booking have been routinely recorded in Scotland for a number of years. This is not yet the case in England and Wales. The data can be linked to NRS civil registration data, which provides an estimate of completeness and contributes further information such as social class. The results will be generalisable to the rest of the UK and internationally. If the HiP grant proved beneficial in Scotland then there is every good reason to believe that a similar impact on outcomes could be achieved elsewhere and certainly in countries with similar health systems and comparable circumstances. Likewise, if the intervention was found to have been more effective for specific subgroups then we might expect subgroups to show greater benefits in other settings.

Study variables

Individual level and area level variables will be used in this study.

Individual level variables will include: birthweight, date of birth, sex, gestational age at delivery, preterm (delivery before the 37th week of pregnancy), weight-for-dates, 5 min Apgar score, crown to heel length, and head circumference. We will distinguish between spontaneous pre-term births and induced pre-term births. A potential reason for induced pre-term births is evidence of poor fetal growth; a proportion of these babies would become more severely growth retarded (more extremely small for gestational age) or stillborn.

Since maternal factors influence fetal growth,[11] individual level variables related to the mother will be examined: parity, age, height, weight at booking, diabetes, smoking status, gestation at booking, booking before 25 weeks, and marital status. Individual socioeconomic position will also be included using data from the birth registrations at NRS. NRS collects occupation for both father and mother for births registered to married couples and jointly

For peer review only - http://bmjopen.bmj.com/site/about/guidelines.xhtml

registered by unmarried couples. Only mother's occupation is recorded for sole registered births. The National Statistics Socio-economic Classification (NS-SEC)[40] will be used to classify the individual socioeconomic position.

Marital status is an important variable as single mothers have consistently been shown to have poorer birth outcomes.[17 19] We are particularly interested in single mothers and social class. Social class for lone mothers is an amalgamation of socioeconomic position and lone parenthood.

Although the routinely collected data on ethnicity are incomplete and of dubious quality, ethnicity remains important for birthweight and other neonatal outcomes.[41-42] We will therefore, within the constraints of the data, include ethnicity and undertake all analyses on the subgroup of mother from a minority ethnic background. Within this subgroup, we will examine the possibility of further distinction between ethnic groups.

Birthweight varies according to the socioeconomic status of the area of residence.[20] The Scottish Index of Multiple Deprivation (SIMD) will be used and included as area level variables in the analysis. The SIMD is the Scottish Government's official tool for identifying those places in Scotland suffering from deprivation.[43] It is a weighted sum of different domains: income; employment; health; education; housing and geographical access (and crime, added in the SIMD 2012). The SIMD provides a comprehensive picture of material deprivation in small areas within Scotland. The index ranks 6505 areas from the most deprived to the least deprived and measures the degree of deprivation of an area relative to that of other areas. The areas employed by the SIMD are datazones and are small: the 6505 datazones have a mean population of 780 people. The reason for employing small area geography at this scale is to permit identification of relatively small pockets of deprivation. The health domain includes an indicator of the proportion of live singleton births that is LBW. The outcomes of this project include birthweight and LBW and so it would not be appropriate to use the health domain or the composite index which includes the health domain. The income domain will therefore be used to assess inequalities at the area level. This domain contributes 28% to the overall index and is highly correlated with the overall SIMD. The income domain of the SIMD identifies areas where there are concentrations of individuals and families living on low incomes. This is done by looking at the numbers of people, both adult and children, who are receiving, or are dependent on, benefits related to

For peer review only

For peer review only - http://bmjopen.bmj.com/site/about/guidelines.xhtml

income or tax credits. Each mother will be assigned to a datazone and its income domain through her home postcode. Previous studies investigating inequalities in birthweight have shown that area deprivation performs as well as or better than individual social class in describing the extent of inequalities in the population.[44-45] However, Fairley et al. who studied the influence of both individual and area based socioeconomic status on temporal trends in Caesarean sections between 1980 and 2000 in Scotland found that maternal social class and area deprivation are different indicators of socioeconomic status which exhibit independent effects on the probability of a woman receiving a Caesarean section.[46] The multilevel analysis will allow us to analyse the effect of both parental social class and SMID and the effect of their interaction on birthweight.

We will adjust the analyses on the urban or rural status of the mother's area of residence. Indeed, Kent et al reported higher adverse birth outcome rates in isolated rural and more population dense areas.[47] They showed that these disparities are being maintained or increasing over time in Alabama. Shankardass et al also found that the patterns of association between socioeconomic position and LGA, spontaneous preterm birth and perinatal death varied depending on urbanicity in Nova Scotia (Canada).[48]

Outcome measures

Primary Outcome

The primary outcome will be birthweight among singleton births. This is influenced by many factors including maternal nutrition and one of the intentions of the HiP grant was to improve this.

Secondary Outcomes

The following secondary outcomes will be assessed:

- Gestation at booking,
- Booking before 25 weeks,
- Measures of size: crown to heel length, head circumference,
- Measures of stage: gestational age at delivery, weight-for-dates (standardised; small for gestational age babies are those weighted less than the 5th centile weight, or large

For peer review only - http://bmjopen.bmj.com/site/about/guidelines.xhtml

for gestational age weighted more than the 90th centile weight), term at birth (pre-term, babies are born at less than 37 weeks gestation; term babies are those born between 37-42 weeks gestation and post-term babies are born after 42 weeks gestation),

- Birth outcomes: mode of delivery, stillbirths, neonatal deaths, 5 minutes Apgar score. Although there is some debate concerning the robustness of the Apgar score as an outcome, it is in common use and we will therefore present results for this outcome within the context of the wide debate on the subject.

Due in part to the introduction of smoking ban in Scotland in 2006, an additional outcome of interest is maternal smoking. Maternal smoking is collected at booking and during pregnancy. The health advice given when receiving the HiP grant might have an impact on smoking rates during pregnancy over and above that of the smoke-free legislation. We will analyse the temporal change in smoking rate by socioeconomic class and its effect on outcomes.

Sample size

The data are clustered in small areas, 6505 datazones. The sample size calculation takes this clustering into account. Assuming an average of 56,000 singleton live births per year, and allowing for the clustering within the 6505 datazones in Scotland (average population 780 per datazone) with an estimated intraclass correlation coefficient of 0.05, we have power of 0.90 to detect an effect of 7g change in birthweight at a 95% significance level. This is not to say that 7g is a clinically important threshold; rather, it is indicative of the power of the study. The large national data set available to us will allow for subgroup analysis. In the 20% most deprived areas, we will have power of 0.80 to detect an effect of 13g; among the 26% of single mothers, we will have power of 0.80 to detect an effect of 11g. To put these small effects into context, 50g is the estimated mean birthweight reduction reported in the meta-analysis of the effect of interventions in pregnancy on maternal and obstetric outcomes.[27]

We anticipate item non-response for some outcomes and explanatory variables. Our primary outcome measure, birthweight, has a completion rate of 99.9%. There is high completion rate (<1.5% missing) for all obstetric variables, with the exception of crown to heel length (15% missing) and head circumference (12% missing). The item non-response ranges from 8% for

For peer review only - http://bmjopen.bmj.com/site/about/guidelines.xhtml

maternal smoking to 20% for marital status. We will use multiple imputation to account for missing data and all analyses will compare the results of analyses of complete cases and multiple imputation. Imputed models will be constructed such that they contain as many relevant predictor variables as possible. The more variables that are used the greater the amount of information available on which estimations are made. We will use all (or as many as possible) obstetric and maternal variables in an imputation model to predict the missing values. It is difficult to identify in advance the number of multiple imputed datasets we will need to construct but it is likely to be between 5 and 10. We will then analyse these datasets identically and combine the results to get the estimates and standard errors for the multiple imputed data. These results will be compared to the complete case analysis results.

It is difficult to be specific about the missing data mechanism until we see the data but much is likely to be missing completely at random (MCAR, e.g. certain hospitals are less likely to collect specific items) or missing at random (MAR, when the missingness is related to known variables and, conditional on these, is assumed to be unrelated to unmeasured variables).

Data analysis plan

Statistical analysis

Descriptive statistics of all variables will be presented as mean, standard deviation, minimum, median and maximum for continuous variables and as proportions when the variables are categorical.

The main statistical design we will use is interrupted time series. Segmented regression analysis will allow an estimation of the size of the effect of the HiP grant at different time points, as well as changes in the trend of the effect over time after its implementation.

Outcomes will be measured on individual births, which are nested within mothers, with mothers themselves clustered within datazones and Health Boards. Multilevel univariable and multivariable models will be used to determine whether the outcomes changed during the period in which the HiP grants were in effect. Multilevel linear regression will be used when the outcome is continuous and multilevel binomial logistic regression when the outcome is dichotomous. Multilevel multinomial logistic regression will be used for the multi-category outcomes mode of delivery and 5 minutes Apgar score.

For peer review only - http://bmjopen.bmj.com/site/about/guidelines.xhtml

All analyses will be adjusted for temporal trends and seasonal variations in outcomes in addition to maternal age, sex of child, parity, marital status, height, weight at booking, diabetes, smoking status, gestation at booking, maternal diabetes, social class, maternal smoking and area deprivation.

The effect of HiP grant on birthweight might have a carryover effect after the withdrawal of the grant. In other words, post-intervention the slope in birthweight might not fall back to the same rate as pre-intervention. This could be due to women who have gave birth during the intervention subsequently having a birth postintervention but still heeding the health advice given during their first pregnancy. We will carry out an additional analysis only on primiparous women to avoid such contamination.

We will analyse pre-term births stratified by mode of delivery and stratified according to whether the birth was induced or spontaneous.

We will repeat the main analyses including (i.e adjusting for the effect of) ethnicity along with other covariates and compare the results with analyses excluding ethnicity to gauge the impact of this on our results. We note that the quality of this variable (including the completeness of recording) is poorer than for other variables and that only 1-2% of mothers delivering in Scotland are from minority ethnic backgrounds.

The simplest model for the intervention effect will include a dummy variable “intervention” covering the period from the introduction to the withdrawal of HiP grants, with adjustment for relevant factors such as marital status. (More complex models of the effect of the intervention will include an interaction of the intervention with the temporal trend). Before carrying out specific subgroup analysis, we will identify differential effects by fitting interaction terms. An assessment as to whether there is a differential effect of the intervention for single women, for example, will involve a test of the significance of the intervention between marital status and the intervention effect. If the interaction is significant this will aid our understanding of the generalisability to other populations, including the rest of the UK. Subgroup analyses will be conducted for those groups seen as having the greatest potential to benefit from the payments such as those living in the most deprived areas, those in the lowest social classes, lone mothers, primiparous women, teen mothers, mothers from ethnic minorities and selected combinations of these groups. For each group we will replicate the main analysis. This reduction in sample size for sub-group analyses will result in fewer

For peer review only - http://bmjopen.bmj.com/site/about/guidelines.xhtml

women/births being available but the same number of areas (datazones) will be analysed (apart from analyses restricted to those living in the most deprived areas).

An increase in birthweight, although desirable at a population level, may not be a beneficial outcome if the baby were already at risk of being large for gestational age (LGA). Separate subgroup analyses will therefore be conducted for women seen to be at high risk of delivering a LGA baby (women with diabetes) and for the remainder of the population. Given that some subgroups may contain small numbers, and bearing in mind the potential importance of the intervention, we will report the results of all subgroup analyses and not just those that reach statistical significance to avoid false negatives. The above process will involve conducting many tests, which will not be independent of each other. Rather than adjusting confidence intervals or p-values to account for this we will present the results of all analyses and caution the user regarding the interpretation of the results. Indeed, some statisticians recommend never correcting for multiple comparisons while analyzing data.[49-50] According to Rothman,[49] reducing the type I error for null associations increases the type II error for those associations that are not null. He recommends a policy of not making adjustments for multiple comparisons because it will lead to fewer errors of interpretation when the data under evaluation are not random numbers but actual observations on nature.

The HiP grant was introduced and withdrawn at the same time as other interventions that may have an impact on birthweight. Healthy Start is a means tested voucher scheme for pregnant women. If they are in receipt of certain benefits or under 18 years old, then they are eligible for free vitamins and vouchers to be spent on fruits and vegetables. This scheme replaced the means tested parts of the Welfare Food Scheme in the UK (including Scotland in 2006) and is still currently in place. During this period, there were policy changes in the optimal timing of first booking appointments due to changes in blood tests offered to pregnant women in the Pregnancy Screening Programme. These changes were first discussed in 2008 and had to be implemented by all Health Boards by March 2011.[51-52] Early booking is a HEAT target of the Scottish Government (Health improvement for the people of Scotland Efficiency and governance improvements, Access to services, Treatment appropriate to individuals H11.1). At least 80% of pregnant women in each SIMD quintile will have booked for antenatal care by the 12th week of gestation by March 2015.

For peer review only - http://bmjopen.bmj.com/site/about/guidelines.xhtml

A further piece of legislation that may affect birthweight is the introduction of the smoking ban in public places in Scotland in March 2006. Mackay et al [53] reported a reduction in the prevalence of current smoking among women who conceived after the introduction of the legislation prohibiting smoking. They also reported a reduction in small and very small for gestational age, as well as in absolute low birth weight after the legislation. We will carry out a further analysis restricting the pre-intervention HiP grant period to April 2006-March 2009.

It is possible that harm may have occurred due to the cash transfer. The £190 was given to pregnant women with no restriction as to how it should be spent, and we do not know how the money was used. We are examining how the intervention group differed; birthweight could have reduced or increased. We will carry out 2-sided hypothesis tests to ensure that we are able to detect any such potential harmful effect.

We will conduct sensitivity analyses to increase the probability that any observed effect can be attributed to the HiP grant. The timing of the HiP grant is well defined and fixed, therefore using the interrupted time series approach any effects within that window can be observed. We plan to carry out 3 analyses for this.

1- We will extend this window for some months before April 2009 (births before the HiP grant was introduced).

2- We will extend this window for some months after April 2011 (births after the HiP grant was withdrawn).

3- We will extend the window both before and after the HiP grant period. In each case we would expect to see a dilution of any effects of the HiP grant.

The statistical analysis plan detailing the outcomes and the covariates, which will be considered for adjustment in the statistical models are presented in table 2.

For peer review only - http://bmjopen.bmj.com/site/about/guidelines.xhtml

Table 2: Analysis plan detailing the outcomes and the covariates that will be considered for adjustment in the statistical models

Covariates	Primary outcome	Secondary outcomes										
		Booking status		Measures of stage			Measures of size	Other birth outcomes				
	**Birth-weight**	Gestation at booking	Booking before 25 weeks	Gestational age at delivery	Term at birth	Weight-for-dates	Head circumference Crown to heel length	Mode of delivery	Still-birth	5 minutes Apgar score	Neonatal death	**Maternal smoking during this pregnancy**
**I- Measured covariates**												
**A- Socio-demographic determinants**												
**A-1. Related to the baby**												
Date of birth	X	X	X	X	X	X	X	X	X	X	X	X
Sex	X			X	X	X	X	X	X	X	X	
Gestational age at delivery	X						X	X	X	X	X	
Birthweight							X			X	X	
Mode of delivery										X	X	
**A-2. Related to the mother**												
Hip grant	X	X	X	X	X	X	X	X	X	X	X	X
Age	X	X	X	X	X	X	X	X	X	X	X	X
Weight at booking	X	X	X	X	X	X	X	X	X	X	X	
Height	X			X	X	X	X	X	X	X	X	
Ethnic group	X	X	X	X	X	X	X	X	X	X	X	X

For peer review only

For peer review only - http://bmjopen.bmj.com/site/about/guidelines.xhtml

Parity	X	X	X	X	X	X	X	X	X	X	X	X
Marital status	X	X	X	X	X	X	X	X	X	X	X	X
Social class	X	X	X	X	X	X	X	X	X	X	X	X
**B- Medical risks of the current pregnancy and before pregnancy**												
Diabetes	X	X	X	X	X	X	X	X	X	X	X	
Hypertension	X	X	X	X	X	X	X	X	X	X	X	
Infection	X			X	X	X	X	X	X	X	X	
Congenital anomalies	X			X	X	X	X	X	X	X	X	
Induction of labour									X	X	X	
Duration of labour									X	X	X	
**C- Medical risks related to previous pregnancies**												
Previous spontaneous abortions		X	X						X			
Previous stillbirths		X	X						X			
Previous neonatal deaths		X	X								X	
**D- Environmental and behavioural risks**												
Income domain of the SIMD	X	X	X	X	X	X	X	X	X	X	X	X
Urban/rural status of the area of residence	X	X	X	X	X	X	X	X	X	X	X	X
Booking status (gestational age at booking, booking before 25 weeks)	X			X	X	X	X	X	X	X	X	X

For peer review only

Smoking status (Before and during pregnancy)	X	X	X	X	X	X	X	X	X	X	X	
Typical weekly alcohol consumption (Before and during pregnancy)	X	X	X	X	X	X	X	X	X	X	X	X
Drug misuse during this pregnancy	X	X	X	X	X	X	X	X	X	X	X	X
**II- Unmeasured covariates**												
Maternal weight gain												
Maternal nutrition												
Maternal education												
Maternal exposure to stress												
Maternal physical activity												
Exposure to toxic substances												
Birth interval												
History of pre-term birth												
**Statistical methods**												
Multilevel linear regression	X	X		X			X					
Multilevel binomial logistic regression			X			X			X		X	X
Multilevel multinomial logistic regression					X			X		X		

For peer review only

X: variables which will be considered for adjustment in the statistical analysis

For peer review only

For peer review only - http://bmjopen.bmj.com/site/about/guidelines.xhtml

Economic analysis

The cost-effectiveness analysis will be based around relating the estimated cost of the intervention (£190 for HiP grant plus the costs of administering the grant) to the observed benefits of the programme (birthweight changes and changes in secondary endpoints such as stillbirths) from the natural experiment. As part of the project an economic model will be developed based on a review of the literature to relate birthweight changes (and any secondary outcomes affected by the HiP grant identified in this study) to long-term cost and health outcomes (in terms of QALYs). Other potential outcomes (such as the effect of birthweight on long term educational outcomes) will be summarised, but may not be included in the cost-per-QALY analysis. The review will inform only the relationship between birthweight and long-term outcomes, the effectiveness of the HiP grant will be taken only from the current study.

The perspective taken will be that of the UK National Health Service in the first instance. For this particular intervention it will be important to consider two further perspectives: the broader Public Sector (due to the relationship between LBW and social care/educational development), and society as whole (since the HiP grant is a transfer payment and therefore there is no net cost to society of transferring the grant from Government to individuals beyond the administration costs).

Of particular interest will be the relative cost-effectiveness of the programme between different socioeconomic groups identified in the main analysis. This may lead to differential policy recommendations for different socioeconomics groups. Uncertainty in the modelling of long-term outcomes will be subject to extensive sensitivity analysis to explore the robustness of the cost-effectiveness analysis.

For peer review only - http://bmjopen.bmj.com/site/about/guidelines.xhtml

IMPLICATIONS

Maternal nutrition plays a crucial role in influencing fetal growth and birth outcomes. It is a modifiable risk factor of public health importance in the effort to prevent adverse birth outcomes, particularly among low-income populations.[24] According to Barker, "*the seeds of inequalities in health in the next century are being sown today, in inner cities and other communities where adverse influences impact upon the growth, nutrition and health of mothers and their infants*".[54]

The HiP grant was cash given to the pregnant women with no constraint on its use. However, economic theory would suggest that cash transfers are more efficient than "vouchers" or subsidies, which try to target the expenditure in the "appropriate expenditure". This is because vouchers, for example, free up disposable income if they displace planned expenditure. This evaluation study may show the HiP grant increased birthweight across the population. If so, then a benefit would be to recommend the re-introduction of a universal cash transfer or, if we believed more evidence was needed that the HiP grants were delivering this benefit, the development of a randomized controlled trial for a similar cash transfer. An additional benefit will be the relative cost-effectiveness of the HiP grant between different socioeconomic groups identified in the main analysis. This may lead to differential policy recommendations for different socioeconomic groups with consequent reduction in health inequalities.

For peer review only - http://bmjopen.bmj.com/site/about/guidelines.xhtml

ETHICS AND DISSEMINATION

Ethical approval is not required as there is no primary data collection. Indeed, the information from maternal and birth records from all hospitals in Scotland are routinely collected. Approval for data collection, storage and release for research purpose has been given (6th May 2014; PAC38A/13) by the Privacy Advisory Committee, an Advisory Committee to NHS National Service Scotland and the Registrar General.

The results of this study will be disseminated through peer-reviewed publications in public health research journals, national and international conferences.

For peer review only

For peer review only - http://bmjopen.bmj.com/site/about/guidelines.xhtml

AUTHOR'S CONTRIBUTIONS

Ruth Dundas was involved in the conception of the study design, literature search and prepared the first draft of the study protocol. She will contribute to the statistical analysis and the interpretation of the results.

Samiratou Ouédraogo contributed to the literature search and the preparation of the present protocol paper. She will be responsible for the statistical analysis and contribute to the interpretation of the results.

Lyndal Bond was involved in the conception of the study design. She will contribute to the interpretation of the results.

Andrew H Briggs designed the health economic analysis aspect of the protocol. He will oversee the cost-effectiveness analysis and assist with writing the health economic paper.

James Chalmers was involved in the conception of the study design.

Ron Gray was involved in the conception of the study design. He will contribute to the interpretation of the results.

Rachael Wood will contribute to the interpretation of the results.

Alastair H Leyland was involved in the conception of the study design, literature search and contributed to all sections. He will contribute to the statistical analysis and the interpretation of the results.

All authors read and approved the final manuscript.

For peer review only

COMPETING INTERESTS

We declare that we have no conflicts of interest.

For peer review only

For peer review only - http://bmjopen.bmj.com/site/about/guidelines.xhtml

FUNDING STATEMENT

This project was funded by the National Institute for Health Research Public Health Research Board (project number 12/3070/02). The Social and Public Health Sciences Unit is core funded by the Medical Research Council (MC_UU_12017/5) and the Chief Scientist Office of the Scottish Government Health Directorates (SPHSU2).

The views and opinions expressed therein are those of the authors and do not necessarily reflect those of the Public Health Research Board, NIHR, NHS or the Department of Health.

For peer review only

For peer review only - http://bmjopen.bmj.com/site/about/guidelines.xhtml

REFERENCE LIST

1. Frankel S, Elwood P, Sweetnam P, et al. Birthweight, body-mass index in middle age, and incident coronary heart disease. Lancet 1996;348(9040):1478-80.

2. Leon DA, Lithell HO, Vagero D, et al. Reduced fetal growth rate and increased risk of death from ischaemic heart disease: cohort study of 15 000 Swedish men and women born 1915-29. BMJ 1998;317(7153):241-5.

3. Reyes L, Manalich R. Long-term consequences of low birth weight. Kidney Int Suppl 2005(97):S107-11.

4. Class QA, Rickert ME, Lichtenstein P, et al. Birth weight, physical morbidity, and mortality: a population-based sibling-comparison study. Am J Epidemiol 2014;179(5):550-8.

5. Hendryx M, Luo J, Knox SS, et al. Identifying multiple risks of low birth weight using person-centered modeling. Womens Health Issues 2014;24(2):e251-6.

6. Barker DJ. Birth weight and hypertension. Hypertension 2006;48(3):357-8.

7. Barker DJ, Eriksson JG, Forsen T, et al. Fetal origins of adult disease: strength of effects and biological basis. Int J Epidemiol 2002;31(6):1235-9.

8. Barker DJ. In utero programming of chronic disease. Clin Sci (Lond) 1998;95(2):115-28.

9. Barker DJ. The fetal and infant origins of adult disease. BMJ 1990;301(6761):1111.

10. Negrato C, Gomes M. Low birth weight: causes and consequences. Diabetol Metab Syndr 2013;5(1):49.

11. Valero De Bernabe J, Soriano T, Albaladejo R, et al. Risk factors for low birth weight: a review. Eur J Obstet Gynecol Reprod Biol 2004;116(1):3-15.

For peer review only - http://bmjopen.bmj.com/site/about/guidelines.xhtml

12. EURO-PERISTAT. “Babies’ health: mortality and morbidity during pregnancy and in the first year of life” in “The European Perinatal Health Report 2010”. 2013. http://www.europeristat.com/images/doc/Peristat%202013%20V2.pdf. (Accessed 28 August 2014)

13. World Health Organisation. International Staticatical Classification of Diseases and Related Health Problems- ICD-10. 10th Revision. Geneva: World Health Organisation, 2010. http://www.who.int/classifications/icd/en/. (Accessed 28 August 2014)

14. OECD. “Infant health: Low birth weight”, in Health at a Glance: Europe 2012. In: Publishing O, ed., 2012. http://www.oecd-ilibrary.org/docserver/download/8112121e.pdf?expires=1404136197&id=id&accname=guest&checksum=368A07F542E03A0442EFDEB9B002B6AE (Accessed 28 August 2014)

15. Macfalane A, Dattani N. European Perinatal Health Report: highlights from a United Kingdom perspective. London, 2013. http://www.europeristat.com/images/Euro-peristat%20UK%20briefing.pdf. (Accessed 28 August 2014)

16. Madden D. The relationship between low birth weight and socioeconomic status in Ireland. J Biosoc Sci 2014;46(2):248-65.

17. Frimmel W, Pruckner GJ. Birth weight and family status revisited: evidence from Austrian register data. Health Econ 2014;23(4):426-45.

18. Huxley R. Smoking, birthweight, and mortality across generations. Eur Heart J 2013;34(44):3398-9.

19. Fairley L, Leyland AH. Social class inequalities in perinatal outcomes: Scotland 1980-2000. J Epidemiol Community Health 2006;60(1):31-6.

For peer review only - http://bmjopen.bmj.com/site/about/guidelines.xhtml

20. Weightman AL, Morgan HE, Shepherd MA, et al. Social inequality and infant health in the UK: systematic review and meta-analyses. BMJ Open 2012;2(3) 2012 Jun 14;2(3):e000964.

21. Juarez SP, Merlo J. Revisiting the effect of maternal smoking during pregnancy on offspring birthweight: a quasi-experimental sibling analysis in Sweden. PLoS One 2013;8(4):e61734.

22. Zdravkovic T, Genbacev O, McMaster MT, et al. The adverse effects of maternal smoking on the human placenta: a review. Placenta 2005;26 Suppl A:S81-6.

23. Villar J, Merialdi M, Gulmezoglu AM, et al. Nutritional interventions during pregnancy for the prevention or treatment of maternal morbidity and preterm delivery: an overview of randomized controlled trials. J Nutr 2003;133(5 Suppl 2):1606S-25S.

24. Abu-Saad K, Fraser D. Maternal nutrition and birth outcomes. Epidemiol Rev 2010;32(1):5-25.

25. Wu G, Imhoff-Kunsch B, Girard AW. Biological mechanisms for nutritional regulation of maternal health and fetal development. Paediatr Perinat Epidemiol 2012;26 Suppl 1:4-26.

26. Mason JB, Saldanha LS, Ramakrishnan U, et al. Opportunities for improving maternal nutrition and birth outcomes: synthesis of country experiences. Food Nutr Bull 2012;33(2 Suppl):S104-37.

27. Thangaratinam S, Rogozinska E, Jolly K, et al. Effects of interventions in pregnancy on maternal weight and obstetric outcomes: meta-analysis of randomised evidence. BMJ 2012;344:e2088.

28. Glassman A, Duran D, Fleisher L, et al. Impact of Conditional Cash Transfers on Maternal and Newborn Health. J Health Popul Nutr 2013;4:19.

For peer review only

For peer review only - http://bmjopen.bmj.com/site/about/guidelines.xhtml

29. Sutherland K, Christianson JB, Leatherman S. Impact of targeted financial incentives on personal health behavior: a review of the literature. Med Care Res Rev 2008;65(6 Suppl):36S-78S.

30. Marteau TM, Ashcroft RE, Oliver A. Using financial incentives to achieve healthy behaviour. BMJ 2009;338:b1415.

31. Giles EL, Robalino S, McColl E, et al. The effectiveness of financial incentives for health behaviour change: systematic review and meta-analysis. PLoS One 2014;9(3):e90347.

32. Brownell M, Chartier M, Au W, et al. Evaluation of the Health Baby Program. Winnipeg, MB: Manitoba Centre for Health Policy. 2010.

33. Neil G. Universal to selective. Transformation of the Welfare State: The Silent Surrender of Public Responsibility: Oxford University Press, 2002:20.

34. Mkandawire T. Targeting and Universalism in poverty reduction. . Secondary Targeting and Universalism in poverty reduction. 2005. http://www.unrisd.org/80256B3C005BCCF9/%28httpAuxPages%29/955FB8A594EEA0B0C12570FF00493EAA/$file/mkandatarget.pdf. (Accessed 28 August 2014)

35. Craig P, Cooper C, Gunnell D, et al. Using natural experiments to evaluate population health interventions: new Medical Research Council guidance. J Epidemiol Community Health 2012;66(12):1182-6.

36. Council MR. Using natural experiments to evaluate population health interventions: guidance for producers and users of evidence. http://www.behaviourworksaustralia.org/wp-content/uploads/2012/10/NaturalExperimentsGuidance_MRC-guidance.pdf (Accessed 28 August 2014)

37. Einarsdottir K, Kemp A, Haggar FA, et al. Increase in caesarean deliveries after the Australian Private Health Insurance Incentive policy reforms. PLoS One 2012;7(7):e41436.

For peer review only - http://bmjopen.bmj.com/site/about/guidelines.xhtml

38. Ramsay CR, Matowe L, Grilli R, et al. Interrupted time series designs in health technology assessment: lessons from two systematic reviews of behavior change strategies. Int J Technol Assess Health Care 2003;19(4):613-23.

39. Penfold RB, Zhang F. Use of interrupted time series analysis in evaluating health care quality improvements. Acad Pediatr 2013;13(6 Suppl):S38-44.

40. Statistics OfN. The National Statistics Socio-economic Classification (NS-SEC rebased on the SOC2010). . Secondary The National Statistics Socio-economic Classification (NS-SEC rebased on the SOC2010). http://www.ons.gov.uk/ons/guide-method/classifications/current-standard-classifications/soc2010/soc2010-volume-3-ns-sec--rebased-on-soc2010--user-manual/index.html. (Accessed 28 August 2014)

41. Scholmerich VL, Erdem O, Borsboom G, et al. The association of neighborhood social capital and ethnic (minority) density with pregnancy outcomes in the Netherlands. PLoS One 2014;9(5):e95873.

42. Margerison-Zilko C. The Contribution of Maternal Birth Cohort to Term Small for Gestational Age in the United States 1989-2010: an Age, Period, and Cohort Analysis. Paediatr Perinat Epidemiol 2014;28(4):312-21

43. The Scottish Government. Scottish Index of Multiple Deprivation 2012: A national Statistics publication for Scotland 2012. http://22fa0f74501b902c9f11-8b3fbddfa1e1fab453a8e75cb14f3396.r26.cf3.rackcdn.com/simd_448749_v7_20121217.pdf. (Accessed 28 August 2014)

44. Pattenden S, Dolk H, Vrijheid M. Inequalities in low birth weight: parental social class, area deprivation, and "lone mother" status. J Epidemiol Community Health 1999;53(6):355-8.

45. Spencer N, Bambang S, Logan S, et al. Socioeconomic status and birth weight: comparison of an area-based measure with the Registrar General's social class. J Epidemiol Community Health 1999;53(8):495-8.

For peer review only - http://bmjopen.bmj.com/site/about/guidelines.xhtml

46. Fairley L, Dundas R, Leyland AH. The influence of both individual and area based socioeconomic status on temporal trends in Caesarean sections in Scotland 1980-2000. BMC Public Health 2011;11:330.

47. Kent ST, McClure LA, Zaitchik BF, et al. Area-level risk factors for adverse birth outcomes: trends in urban and rural settings. BMC Pregnancy Childbirth 2013;13:129.

48. Shankardass K, O'Campo P, Dodds L, et al. Magnitude of income-related disparities in adverse perinatal outcomes. BMC Pregnancy Childbirth 2014;14:96.

49. Rothman KJ. No adjustments are needed for multiple comparisons. Epidemiology 1990;1(1):43-6.

50. Greenland S. Multiple comparisons and association selection in general epidemiology. Int J Epidemiol 2008;37(3):430-4.

51. National Service Division. Pregnancy Screening Programmes. http://www.nsd.scot.nhs.uk/services/screening/pregscreening/index.html. (Accessed 28 August 2014)

52. NHS Health Scotland. Your guide to screening tests during pregnancy. http://www.healthscotland.com/uploads/documents/3985-YourGuideToScreeningTestsDuringPregnancy_1.pdf. (Accessed 28 August 2014)

53. Mackay DF, Nelson SM, Haw SJ, et al. Impact of Scotland's smoke-free legislation on pregnancy complications: retrospective cohort study. PLoS Med 2012;9(3):e1001175.

54. Barker DJ. The foetal and infant origins of inequalities in health in Britain. J Public Health Med 1991;13(2):64-8.

For peer review only - http://bmjopen.bmj.com/site/about/guidelines.xhtml

BMJ Open

Evaluation of Health in Pregnancy grants in Scotland: a protocol for a natural experiment

Journal:	*BMJ Open*
Manuscript ID:	bmjopen-2014-006547.R1
Article Type:	Protocol
Date Submitted by the Author:	23-Sep-2014
Complete List of Authors:	Dundas, Ruth; University of Glasgow, MRC/CSO Social and Public Health Sciences Unit<br>Ouédraogo, Samiratou; University of Glasgow, MRC/CSO Social and Public Health Sciences Unit<br>Bond, Lyndal; Centre of Excellence in Intervention and Prevention Science,<br>Briggs, Andrew; University of Glasgow, Health Economics & Health Technology Assessment Institute of Health & Wellbeing<br>Chalmers, James; NHS National Services Scotland, Information Services Division<br>Gray, Ron; University of Oxford, National Perinatal Epidemiology Unit<br>Wood, Rachael; NHS National Services Scotland, Information Services Division<br>Leyland, Alastair; University of Glasgow, MRC/CSO Social and Public Health Sciences Unit
**Primary Subject Heading**:	Health policy
Secondary Subject Heading:	Epidemiology, Public health, Health economics, Research methods
Keywords:	EPIDEMIOLOGY, Reproductive medicine < GYNAECOLOGY, HEALTH ECONOMICS, Health policy < HEALTH SERVICES ADMINISTRATION & MANAGEMENT, NEONATOLOGY, Maternal medicine < OBSTETRICS

SCHOLARONE™
Manuscripts

PROJECT PROTOCOL

Title: Evaluation of Health in Pregnancy grants in Scotland: a protocol for a natural experiment

Authors: Ruth Dundas[1], Samiratou Ouédraogo[1], Lyndal Bond[2], Andrew H Briggs[3], James Chalmers[4], Ron Gray[5], Rachael Wood[4], Alastair H Leyland[1]

Affiliations:

[1] MRC/CSO Social and Public Health Sciences Unit, University of Glasgow, Glasgow, UK

[2] Centre of Excellence in Intervention and Prevention Science, Melbourne, Australia

[3] Health Economics and Health Technology Assessment, University of Glasgow, Glasgow, UK

[4] Information Services Division, NHS National Services Scotland, Edinburgh, UK

[5] National Perinatal Epidemiology Unit, University of Oxford, UK

Correspondence to:

Ruth Dundas

MRC/CSO Social and Public Health Sciences Unit, University of Glasgow
200 Renfield Street, Glasgow G2 3QB, Scotland
Tel: +44 141 353 7541 Email: ruth.dundas@glasgow.ac.uk

Keywords: Low birthweight; interrupted time series; multilevel analysis; Health in Pregnancy grant; natural experiment.

For peer review only

ABSTRACT

Introduction

A substantial proportion of low birthweight (LBW) is attributable to the mother's cultural and socioeconomic circumstances. Early childhood programmes have been widely developed to improve child outcomes. In the UK, the Health in Pregnancy (HiP) grant, a universal conditional cash transfer of £190, was introduced for women reaching the 25th week of pregnancy with a due date on/or after 6th April 2009 and subsequently withdrawn for women reaching the 25th week of pregnancy on/or after 1st January 2011. The current study focuses on the evaluation of the effectiveness and cost-effectiveness of the HiP grant.

Methods and analysis

The population under study will be all singleton births in Scotland over the periods of January 2004 to March 2009 (pre-intervention), April 2009 to April 2011 (intervention) and May 2011 to December 2013 (post-intervention). Data will be extracted from the Scottish maternity and neonatal database. The analysis period 2004-2013 should yield over 585,000 births. The primary outcome will be birthweight among singleton births. Other secondary outcomes will include gestation at booking, booking before 25 weeks; measures of size and stage; gestational age at delivery; weight-for-dates, term at birth; birth outcomes and maternal smoking. The main statistical method we will use is interrupted time series. Outcomes will be measured on individual births nested within mothers, with mothers themselves clustered within datazones. Multilevel regression models will be used to determine whether the outcomes changed during the period in which the HiP grants was in effect. Subgroup analyses will be conducted for those groups most likely to benefit from the payments.

Ethics and dissemination

Approval for data collection, storage and release for research purpose has been given (6th of May 2014, PAC38A/13) by the Privacy Advisory Committee. The results of this study will be disseminated through peer-reviewed publications in journals, national and international conferences.

For peer review only

For peer review only - http://bmjopen.bmj.com/site/about/guidelines.xhtml

ARTICLE SUMMARY

Article focus

- To evaluate the effectiveness and cost-effectiveness of Health in Pregnancy grant (HiP) in Scotland.

Key messages

- We will assess the difference in birthweight for babies born to those mothers who were eligible for the HiP grant with babies born before the HiP grant was introduced or after it was withdrawn using interrupted time series multilevel analysis.
- Subgroup analyses will be conducted for those groups seen as having the greatest potential to benefit from the payments such as those living in the most deprived areas, those in the lowest social classes, lone mothers, primiparous women, teen mothers, mothers from ethnic minorities and selected combinations of these groups.
- As part of the project an economic model will be developed based on a review of the literature to relate birthweight changes to long-term cost and health outcomes (in terms of QALYs).

Strengths and limitations of this study

The strengths of this study are:

- This is the first study evaluating the effect and the cost-effectiveness of the HiP grant in Scotland. It will use routinely available vital event and maternal and neonatal health records that are known to have high completeness and accuracy.
- The evaluation of the HiP grant using an interrupted time series design will enable us to analyse birthweight trends in Scotland and detect whether the intervention has had an effect over and above the underlying temporal trend. The use of interrupted time series will overcome other biases such as the autocorrelation of repeated measurements (measurement taken close together are related), seasonal effects (birthweight varies according to month of birth), the duration of the intervention (we will have pre-intervention intervention and post-intervention), and random variation in the measurement (birthweight).

The limitations of this study

For peer review only

For peer review only - http://bmjopen.bmj.com/site/about/guidelines.xhtml

- This study will evaluate the effect and the cost-effectiveness of the HiP grant, birthweight and other birth outcomes in Scotland using obstetric as well as maternal data. However, these outcomes may be influenced by many factors, not all of which are routinely captured (such as maternal diet, maternal work and psychological stress, abuse, exposure to toxic substance).[11]
- The HiP grant was money given to pregnant women with no constraint on its use. The use of routine data gives us no indication on how the money was spent.

For peer review only

For peer review only - http://bmjopen.bmj.com/site/about/guidelines.xhtml

INTRODUCTION

Low birthweight (LBW) due to preterm birth, intrauterine growth restriction, or being born small for gestational age (SGA), is commonly associated with perinatal mortality and impaired development[1-11]. Despite the improvement of the mortality in LBW babies over the past three decades, more than 70% of all neonatal mortality in Europe is found in infants weighing less than 2500 grams (g).[12]

LBW refers to birth weights below 2500 g,[13] irrespective of the gestational age of the infant. In most developed countries, the prevalence of LBW has increased due to several reasons: the number of multiple births with the increased risks of pre-term births and low birth weight partly as a result of the rise in fertility treatments; older age at childbearing and increases in the use of delivery management techniques such as induction of labour and caesarean delivery, which have increased the survival rates of low birth weight babies.[14] About one in 20 babies born in Europe in 2010 weighed less than 2500 g at birth.[12] With LBW estimated at 7.0% of live births in England and Wales, 6.5% in Scotland and 5.7% of live births in Northern Ireland, prevalence of LBW in the countries of the United Kingdom (UK) tended to be higher than in the rest of Europe.[12-15] Moreover, compared with other Western European countries, the UK has an incidence of LBW (<2500g) and very LBW (<1500g) in the top third. The proportion of pre-term birth (<37 weeks) is also high compared with other Western European countries.

Considerable attention has been focused on the causal determinants of LBW, in order to identify potentially modifiable factors. A substantial proportion of LBW is attributable to the mother's cultural and socioeconomic circumstances such as socioeconomic status (SES), harmful behaviours (smoking and excessive alcohol consumption) and poor nutrition during pregnancy.[11,16-18] In a study of social class inequalities in perinatal outcomes in Scotland, Fairley and Leyland[19] reported a percentage of 5.8% LBW in unskilled social class (V) compared to 2.9% in professional social class (I) between 1995 and 2000. The systematic review and meta-analyses of social inequality and infant health in the UK performed by Weightman et al.[20] found that the odds ratio for low birth weight was 1.79 (95% CI 1.43 to 2.24) in the lowest compared to the highest social class. This effect may vary with maternal factors such as age and smoking status. Smoking during pregnancy reduces birthweight by 162 to 377 g, depending on daily consumption (larger reduction for heavy smokers) and the trimester in which exposure occurs (larger reduction during the last trimester).[18,21-22]

For peer review only - http://bmjopen.bmj.com/site/about/guidelines.xhtml

The association between maternal nutrition and birth outcome is complex and is influenced by many biologic, socioeconomic, and demographic factors, which vary widely in different populations.[23-24] However, it has been reported that favourable prenatal nutrition associated with adequate prenatal care can have a positive impact on birth outcomes and morbidity in adult life.[25-26] Indeed, the developmental model of the origins of chronic disease proposes the causal influence of undernutrition *in utero* on coronary heart disease and stroke in adult life.[7-9] An improvement in fetal nutrition may therefore have far reaching consequences in terms of the prevention of disease. A review of maternal nutrition and birth outcomes identified improving maternal nutrition as being beneficial to the prevention of adverse birth outcomes in lower social class groups.[24]

A number of early childhood programmes have been developed to improve child outcomes. There is mixed evidence that these programmes do provide such improvement. In a meta-analysis of the effect of interventions in pregnancy on maternal and obstetric outcomes, Thangaratinam et al. concluded that dietary and lifestyle interventions in pregnancy reduced maternal gestational weight gain but had less effect on outcomes related to fetal weight and other morbidity and mortality.[27] Glassman et al.[28] who reported the results of conditional cash transfer programmes increasingly being adopted and scaled in developing countries, found that the programmes have increased the uptake of maternal and newborn health services, especially skilled attendance at delivery and antenatal monitoring. However, the impact of the programs on maternal and newborn mortality has not been well documented. Therefore, they recommended more rigorous impact evaluations that document impact pathways and take factors, such as cost-effectiveness, into account. Other studies evaluating payments to influence health behaviour found financial incentives were effective in increasing infrequent behaviours such as attending clinic appointments particularly in low-income groups, and recommended payments as being more effective than information and less restrictive than legislation.[29-31] In Canada, Brownell et al. have evaluated a complex programme on a prenatal benefit provided to families on low income during pregnancy.[32] They found that the receipt of this prenatal benefit was associated with a reduction in incidence of both low birthweight babies and preterm births. They suggested that efforts should be made to ensure all low-income women receive the income supplement.

In the UK, the Health in Pregnancy (HiP) grant was introduced for women reaching the 25th week of pregnancy with a due date on/or after 6th April 2009. It was subsequently withdrawn for women reaching the 25th week of pregnancy on/or after 1st January 2011. The HiP grant

For peer review only - http://bmjopen.bmj.com/site/about/guidelines.xhtml

was a universal conditional cash transfer of £190 for women reaching 25 weeks of pregnancy if they had sought health advice from a doctor or midwife. It was designed to provide additional financial support, in the last months of pregnancy, towards a healthy lifestyle including diet, and it was suggested that the link to the requirement for pregnant women to seek health advice from a professional may provide a greater incentive for expectant mothers to seek the recommended health advice at the appropriate time. The grant was paid and administered by Her Majesty's Revenue and Customs (HMRC) on receipt of a claim form partly completed by the midwife or doctor. Advice was offered as normal by doctors and midwives. Payment was made directly into a bank account with a telephone helpline available to provide support through the claims process including options for payment in the event of difficulties opening a bank account. Take up of the grant was said to be about the same level as for child benefit (98-99%, personal communication, HMRC).

The current study focuses on the evaluation of the effectiveness and cost-effectiveness of the HiP grant. As a primary outcome, we will consider the difference in birthweight for babies born to those mothers who were eligible for the HiP grant with babies born before the HiP grant was introduced or after it was withdrawn. Specific questions the research project will address are:

- Were there differential impacts of the intervention for particular subgroups defined by socioeconomic (defined in terms of both area deprivation and individual occupational social class), demographic (marital status, age, maternal height), or obstetric (parity, previous caesarean section) factors or for selected combination of these groups?
- Was the HiP grant cost-effective? How did cost-effectiveness vary across important subgroups identified as having differential outcomes?

The principle of universalism in the allocation of social benefits, that is the availability of social benefits to everyone as of right, is contrasted with allocation on a selective basis (targeted) in which benefits are allocated on the basis of need as determined by means testing of income.[33] The advantage of universal benefits is that they are easy to administer and can be efficiently delivered. The major disadvantage is that they are expensive, because they are delivered to those who do not need them as much as to those who do. However, the use of targeting involves some mechanism that discriminates between the poor and the non-poor. As such it always runs the danger of committing either type I errors which occur when someone

For peer review only - http://bmjopen.bmj.com/site/about/guidelines.xhtml

who deserves the benefits is denied them (underpayment, false positives), or type II errors, which occur when benefits are paid to someone who does not deserve them (overpayment, leakage).[34] The HiP grant represented an attempt to influence behaviour – appropriate and timely receipt of antenatal care advice – by means of a relatively modest, universally applied cash transfer. The evaluation of the effectiveness of such a payment may inform other policies aiming to change behaviour.

For peer review only

For peer review only - http://bmjopen.bmj.com/site/about/guidelines.xhtml

METHODS AND ANALYSIS

Study design

The HiP grant will be evaluated as a natural experiment using interrupted time series analysis to compare outcomes before the introduction of the intervention in Scotland and immediately after its withdrawal with those during the period for which it existed.

The Medical Research Council (MRC) has issued guidelines regarding the use of natural experiments to evaluate population health interventions when exposure to the intervention has not been manipulated by the researchers (Table 1).[35-36]

Table 1: Guidance for use of natural experiments to evaluate population health interventions

When to use a natural experimental approach?	How does the evaluation of HiP* grant meet these criteria?
▪ There is a reasonable expectation that the intervention will have a significant health impact, but scientific uncertainty about the size or nature of the effects.	▪ The HiP grant represented an attempt to influence behaviour – appropriate and timely receipt of antenatal care advice. With the sample size we are using in this evaluation study, we are able to detect small changes in birthweight.
▪ Natural experimental study is the most appropriate method for studying a given type of intervention.	▪ The HiP grant was a universally applied cash transfer available for all pregnant women with no discrimination between socioeconomic classes. This policy was not introduced using a randomised allocation
▪ It is possible to obtain the relevant data from an appropriate study population, comprising groups with different levels of exposure to the intervention.	▪ The uptake of the HiP grant was thought to be 98-99%. The linked Scottish birth dataset has 98% coverage of births and the primary outcome, birthweight, is well measured, 99.9% complete and accurate. Exposure is determined by the dates for which the HiP grant was in existence.
▪ The intervention or the principles behind it have the potential for replication, scalability or generalisability.	▪ The HiP grant is replicable everywhere in countries with similar health systems.

HiP* grant: Health in Pregnancy grant

For peer review only - http://bmjopen.bmj.com/site/about/guidelines.xhtml

The guidance advocates a number of designs including regression discontinuity designs such as interrupted time series. The interrupted time series approach is a powerful tool used for evaluating the impact of a policy change or quality improvement programme on the rate of an outcome in a defined population of individuals.[37-39] This approach will allow us to use a comparison group of pregnant women who delivered before the HiP grant was introduced, an intervention group who received the HiP grant and an additional post-intervention group who delivered after the HiP grant was withdrawn. It will also allow adjustment for seasonality, temporal trends and the socio-demographic and obstetric characteristics of the mother.

Study population

The population under study will be all singleton births in Scotland over the periods of January 2004 to March 2009 (pre-intervention), April 2009 to April 2011 (intervention) and May 2011 to December 2013 (post-intervention).

The Scottish maternity and neonatal database is a comprehensive record linkage system.[40-41] Probabilistic linkage procedures are used to add a unique identifier to all datasets to ensure all relevant records relating to an individual can be linked as required. It facilitates the linkage of a number of records from the system of Scottish Morbidity Records (SMR) including mother's obstetric records (SMR02) and the baby's birth and neonatal information from Scottish Birth Records (SBR).[42] Further links to the stillbirth and infant Death Survey and the National Records of Scotland (NRS) birth, stillbirth and infant death records can be carried out. The coverage is almost all births in Scotland.[43] From NRS data, we know of all registered births in Scotland (ie 100%). We have suitable record linked data on 98% of these births.[44] These will nearly all be hospital births; a fairly high proportion of the missing records are home births.

There is an average of about 56,000 births per year in Scotland. [45] The analysis period 2004-2013 should yield over 585,000 births. The analysis period 2006-2013 should yield over 455,000 births.

For peer review only - http://bmjopen.bmj.com/site/about/guidelines.xhtml

We have chosen to use data from the Scotland for this evaluation for the following reasons. First, data are available at a national level on the approximately 56,000 deliveries per year. Second, Scotland has a long history of collecting high quality routine data. The coverage, completeness and quality of the data are considered to be very high.[46] Third, the concentration of deprivation within parts of Scotland is unique within the UK.[47] And using a UK wide Cartstairs index, the Scotland's population is over-represented in the bottom 5 deciles compared to England.[48] Fourth, data on smoking at booking have been routinely recorded in Scotland for a number of years.[49] This is not yet the case in England and Wales. The data can be linked to NRS civil registration data, which provides an estimate of completeness and contributes further information such as social class. The results will be generalisable to the rest of the UK and internationally. If the HiP grant proved beneficial in Scotland then there is every good reason to believe that a similar impact on outcomes could be achieved elsewhere and certainly in countries with similar health systems and comparable circumstances. Likewise, if the intervention was found to have been more effective for specific subgroups then we might expect subgroups to show greater benefits in other settings.

Study variables

Individual level and area level variables will be used in this study.

Individual level variables will include: birthweight, date of birth, sex, gestational age at delivery, preterm (delivery before the 37th week of pregnancy), weight-for-dates, 5 min Apgar score, crown to heel length, and head circumference. We will distinguish between spontaneous pre-term births and induced pre-term births. A potential reason for induced pre-term births is evidence of poor fetal growth; a proportion of these babies would become more severely growth retarded (more extremely small for gestational age) or stillborn.

Since maternal factors influence fetal growth,[11] individual level variables related to the mother will be examined: parity, age, height, weight at booking, diabetes, smoking status, gestation at booking, booking before 25 weeks, and marital status. Individual socioeconomic position will also be included using data from the birth registrations at NRS.[50] NRS collects occupation for both father and mother for births registered to married couples and jointly registered by unmarried couples.[51] Only mother's occupation is recorded for sole registered

For peer review only - http://bmjopen.bmj.com/site/about/guidelines.xhtml

births. The National Statistics Socio-economic Classification (NS-SEC)[52] will be used to classify the individual socioeconomic position.

Marital status is an important variable as single mothers have consistently been shown to have poorer birth outcomes.[17-19] We are particularly interested in single mothers and social class. Social class for lone mothers is an amalgamation of socioeconomic position and lone parenthood.

Although the routinely collected data on ethnicity are incomplete and of dubious quality,[46,53] ethnicity remains important for birthweight and other neonatal outcomes.[54-55] We will therefore, within the constraints of the data, include ethnicity and undertake all analyses on the subgroup of mother from a minority ethnic background. Within this subgroup, we will examine the possibility of further distinction between ethnic groups.

Birthweight varies according to the socioeconomic status of the area of residence.[20] The Scottish Index of Multiple Deprivation (SIMD) will be used and included as area level variables in the analysis. The SIMD is the Scottish Government's official tool for identifying those places in Scotland suffering from deprivation.[56] It is a weighted sum of different domains: income; employment; health; education; housing and geographical access (and crime, added in the SIMD 2012). The SIMD provides a comprehensive picture of material deprivation in small areas within Scotland. The index ranks 6505 areas from the most deprived to the least deprived and measures the degree of deprivation of an area relative to that of other areas. The areas employed by the SIMD are datazones and are small: the 6505 datazones have a mean population of 780 people. The reason for employing small area geography at this scale is to permit identification of relatively small pockets of deprivation. The health domain includes an indicator of the proportion of live singleton births that is LBW. The outcomes of this project include birthweight and LBW and so it would not be appropriate to use the health domain or the composite index which includes the health domain. The income domain will therefore be used to assess inequalities at the area level. This domain contributes 28% to the overall index and is highly correlated with the overall SIMD. The income domain of the SIMD identifies areas where there are concentrations of individuals and families living on low incomes. This is done by looking at the numbers of people, both adult and children, who are receiving, or are dependent on, benefits related to income or tax credits.[57] Each mother will be assigned to a datazone and its income domain

For peer review only - http://bmjopen.bmj.com/site/about/guidelines.xhtml

through her home postcode. Previous studies investigating inequalities in birthweight have shown that area deprivation performs as well as or better than individual social class in describing the extent of inequalities in the population.[58-59] However, Fairley et al. who studied the influence of both individual and area based socioeconomic status on temporal trends in Caesarean sections between 1980 and 2000 in Scotland found that maternal social class and area deprivation are different indicators of socioeconomic status which exhibit independent effects on the probability of a woman receiving a Caesarean section.[60] The multilevel analysis will allow us to analyse the effect of both parental social class and SMID and the effect of their interaction on birthweight.

We will adjust the analyses on the urban or rural status of the mother's area of residence. Indeed, Kent et al reported higher adverse birth outcome rates in isolated rural and more population dense areas.[61] They showed that these disparities are being maintained or increasing over time in Alabama. Shankardass et al also found that the patterns of association between socioeconomic position and LGA, spontaneous preterm birth and perinatal death varied depending on urbanicity in Nova Scotia (Canada).[62]

Outcome measures

Primary Outcome

The primary outcome will be birthweight among singleton births. This is influenced by many factors including maternal nutrition and one of the intentions of the HiP grant was to improve this.

Secondary Outcomes

The following secondary outcomes will be assessed:

- Gestation at booking,
- Booking before 25 weeks,
- Measures of size: crown to heel length, head circumference,
- Measures of stage: gestational age at delivery, weight-for-dates (standardised; small for gestational age babies are those weighted less than the 5th centile weight, or large for gestational age weighted more than the 90th centile weight), term at birth (pre-

For peer review only - http://bmjopen.bmj.com/site/about/guidelines.xhtml

term, babies are born at less than 37 weeks gestation; term babies are those born between 37-42 weeks gestation and post-term babies are born after 42 weeks gestation),

- Birth outcomes: mode of delivery, stillbirths, neonatal deaths, 5 minutes Apgar score. Although there is some debate concerning the robustness of the Apgar score as an outcome, it is in common use and we will therefore present results for this outcome within the context of the wide debate on the subject.

Due in part to the introduction of smoking ban in Scotland in 2006, an additional outcome of interest is maternal smoking. Maternal smoking is collected at booking and during pregnancy. The health advice given when receiving the HiP grant might have an impact on smoking rates during pregnancy over and above that of the smoke-free legislation. We will analyse the temporal change in smoking rate by socioeconomic class and its effect on outcomes.

Sample size

The data are clustered in small areas, 6505 datazones. The sample size calculation takes this clustering into account. Assuming an average of 56,000 singleton live births per year, and allowing for the clustering within the 6505 datazones in Scotland (average population 780 per datazone) with an estimated intraclass correlation coefficient of 0.05, we have power of 0.90 to detect an effect of 7g change in birthweight at a 95% significance level. This is not to say that 7g is a clinically important threshold; rather, it is indicative of the power of the study. The large national data set available to us will allow for subgroup analysis. In the 20% most deprived areas, we will have power of 0.80 to detect an effect of 13g; among the 26% of single mothers, we will have power of 0.80 to detect an effect of 11g. To put these small effects into context, 50g is the estimated mean birthweight reduction reported in the meta-analysis of the effect of interventions in pregnancy on maternal and obstetric outcomes.[27]

We anticipate item non-response for some outcomes and explanatory variables. Our primary outcome measure, birthweight, has a completion rate of 99.9%. There is high completion rate (<1.5% missing) for all obstetric variables,[46] with the exception of crown to heel length (15% missing) and head circumference (12% missing). The item non-response ranges from 8% for maternal smoking to 20% for marital status. We will use multiple imputation to account for

For peer review only - http://bmjopen.bmj.com/site/about/guidelines.xhtml

missing data and all analyses will compare the results of analyses of complete cases and multiple imputation.[63] Imputed models will be constructed such that they contain as many relevant predictor variables as possible. The more variables that are used the greater the amount of information available on which estimations are made. We will use all (or as many as possible) obstetric and maternal variables in an imputation model to predict the missing values. It is difficult to identify in advance the number of multiple imputed datasets we will need to construct but it is likely to be between 5 and 10. We will then analyse these datasets identically and combine the results to get the estimates and standard errors for the multiple imputed data. These results will be compared to the complete case analysis results.

It is difficult to be specific about the missing data mechanism until we see the data but much is likely to be missing completely at random (MCAR, e.g. certain hospitals are less likely to collect specific items) or missing at random (MAR, when the missingness is related to known variables and, conditional on these, is assumed to be unrelated to unmeasured variables).

Data analysis plan

Statistical analysis

Descriptive statistics of all variables will be presented as mean, standard deviation, minimum, median and maximum for continuous variables and as proportions when the variables are categorical.

The main statistical design we will use is interrupted time series.[64] Segmented regression analysis will allow an estimation of the size of the effect of the HiP grant at different time points, as well as changes in the trend of the effect over time after its implementation.

Outcomes will be measured on individual births, which are nested within mothers, with mothers themselves clustered within datazones and Health Boards. Multilevel univariable and multivariable models will be used to determine whether the outcomes changed during the period in which the HiP grants were in effect. Multilevel linear regression will be used when the outcome is continuous and multilevel binomial logistic regression when the outcome is dichotomous. Multilevel multinomial logistic regression will be used for the multi-category outcomes mode of delivery and 5 minutes Apgar score.

For peer review only - http://bmjopen.bmj.com/site/about/guidelines.xhtml

All analyses will be adjusted for temporal trends and seasonal variations in outcomes in addition to maternal age, sex of child, parity, marital status, height, weight at booking, diabetes, smoking status, gestation at booking, maternal diabetes, social class, maternal smoking and area deprivation.

The effect of HiP grant on birthweight might have a carryover effect after the withdrawal of the grant. In other words, post-intervention the slope in birthweight might not fall back to the same rate as pre-intervention. This could be due to women who have gave birth during the intervention subsequently having a birth postintervention but still heeding the health advice given during their first pregnancy. We will carry out an additional analysis only on primiparous women to avoid such contamination.

We will analyse pre-term births stratified by mode of delivery and stratified according to whether the birth was induced or spontaneous.

We will repeat the main analyses including (i.e adjusting for the effect of) ethnicity along with other covariates and compare the results with analyses excluding ethnicity to gauge the impact of this on our results. We note that the quality of this variable (including the completeness of recording) is poorer than for other variables and that only 1-2% of mothers delivering in Scotland are from minority ethnic backgrounds.

The simplest model for the intervention effect will include a dummy variable “intervention” covering the period from the introduction to the withdrawal of HiP grants, with adjustment for relevant factors such as marital status. (More complex models of the effect of the intervention will include an interaction of the intervention with the temporal trend). Before carrying out specific subgroup analysis, we will identify differential effects by fitting interaction terms. An assessment as to whether there is a differential effect of the intervention for single women, for example, will involve a test of the significance of the intervention between marital status and the intervention effect. If the interaction is significant this will aid our understanding of the generalisability to other populations, including the rest of the UK. Subgroup analyses will be conducted for those groups seen as having the greatest potential to benefit from the payments such as those living in the most deprived areas, those in the lowest social classes, lone mothers, primiparous women, teen mothers, mothers from ethnic minorities and selected combinations of these groups. For each group we will replicate the main analysis. This reduction in sample size for sub-group analyses will result in fewer

For peer review only

For peer review only - http://bmjopen.bmj.com/site/about/guidelines.xhtml

women/births being available but the same number of areas (datazones) will be analysed (apart from analyses restricted to those living in the most deprived areas).

An increase in birthweight, although desirable at a population level, may not be a beneficial outcome if the baby were already at risk of being large for gestational age (LGA). Separate subgroup analyses will therefore be conducted for women seen to be at high risk of delivering a LGA baby (women with diabetes) and for the remainder of the population. Given that some subgroups may contain small numbers, and bearing in mind the potential importance of the intervention, we will report the results of all subgroup analyses and not just those that reach statistical significance to avoid false negatives. The above process will involve conducting many tests, which will not be independent of each other. Rather than adjusting confidence intervals or p-values to account for this we will present the results of all analyses and caution the user regarding the interpretation of the results. Indeed, some statisticians recommend never correcting for multiple comparisons while analyzing data.[65-66] According to Rothman,[65] reducing the type I error for null associations increases the type II error for those associations that are not null. He recommends a policy of not making adjustments for multiple comparisons because it will lead to fewer errors of interpretation when the data under evaluation are not random numbers but actual observations on nature.

The HiP grant was introduced and withdrawn at the same time as other interventions that may have an impact on birthweight. Healthy Start is a means tested voucher scheme for pregnant women. If they are in receipt of certain benefits or under 18 years old, then they are eligible for free vitamins and vouchers to be spent on fruits and vegetables. This scheme replaced the means tested parts of the Welfare Food Scheme in the UK (including Scotland in 2006) and is still currently in place. During this period, there were policy changes in the optimal timing of first booking appointments due to changes in blood tests offered to pregnant women in the Pregnancy Screening Programme. These changes were first discussed in 2008 and had to be implemented by all Health Boards by March 2011.[67-68] Early booking is a HEAT target of the Scottish Government (Health improvement for the people of Scotland Efficiency and governance improvements, Access to services, Treatment appropriate to individuals H11.1).[69] At least 80% of pregnant women in each SIMD quintile will have booked for antenatal care by the 12th week of gestation by March 2015.

For peer review only - http://bmjopen.bmj.com/site/about/guidelines.xhtml

A further piece of legislation that may affect birthweight is the introduction of the smoking ban in public places in Scotland in March 2006. Mackay et al [70] reported a reduction in the prevalence of current smoking among women who conceived after the introduction of the legislation prohibiting smoking. They also reported a reduction in small and very small for gestational age, as well as in absolute low birth weight after the legislation. We will carry out a further analysis restricting the pre-intervention HiP grant period to April 2006-March 2009.

It is possible that harm may have occurred due to the cash transfer. The £190 was given to pregnant women with no restriction as to how it should be spent, and we do not know how the money was used. We are examining how the intervention group differed; birthweight could have reduced or increased. We will carry out 2-sided hypothesis tests to ensure that we are able to detect any such potential harmful effect.

We will conduct sensitivity analyses to increase the probability that any observed effect can be attributed to the HiP grant. The timing of the HiP grant is well defined and fixed, therefore using the interrupted time series approach any effects within that window can be observed. We plan to carry out 3 analyses for this.

1- We will extend this window for some months before April 2009 (births before the HiP grant was introduced).

2- We will extend this window for some months after April 2011 (births after the HiP grant was withdrawn).

3- We will extend the window both before and after the HiP grant period. In each case we would expect to see a dilution of any effects of the HiP grant.

The statistical analysis plan detailing the outcomes and the covariates, which will be considered for adjustment in the statistical models are presented in table 2.

For peer review only

Table 2: Analysis plan detailing the outcomes and the covariates that will be considered for adjustment in the statistical models

	Primary outcome	Secondary outcomes										
		Booking status		Measures of stage			Measures of size	Other birth outcomes				
Covariates	Birth-weight	Gestation at booking	Booking before 25 weeks	Gestational age at delivery	Term at birth	Weight-for-dates	Head circumference Crown to heel length	Mode of delivery	Still-birth	5 minutes Apgar score	Neonatal death	Maternal smoking during this pregnancy
**I- Measured covariates**												
**A- Socio-demographic determinants**												
**A-1. Related to the baby**												
Date of birth	X	X	X	X	X	X	X	X	X	X	X	X
Sex	X			X	X	X	X	X	X	X	X	
Gestational age at delivery	X						X	X	X	X	X	
Birthweight							X			X	X	
Mode of delivery										X	X	
**A-2. Related to the mother**												
Hip grant	X	X	X	X	X	X	X	X	X	X	X	X
Age	X	X	X	X	X	X	X	X	X	X	X	X
Weight at booking	X	X	X	X	X	X	X	X	X	X	X	
Height	X			X	X	X	X	X	X	X	X	
Ethnic group	X	X	X	X	X	X	X	X	X	X	X	X

For peer review only

For peer review only - http://bmjopen.bmj.com/site/about/guidelines.xhtml

Parity	X	X	X	X	X	X	X	X	X	X	X	X
Marital status	X	X	X	X	X	X	X	X	X	X	X	X
Social class	X	X	X	X	X	X	X	X	X	X	X	X
**B- Medical risks of the current pregnancy and before pregnancy**												
Diabetes	X	X	X	X	X	X	X	X	X	X	X	
Hypertension	X	X	X	X	X	X	X	X	X	X	X	
Infection	X			X	X	X	X	X	X	X	X	
Congenital anomalies	X			X	X	X	X	X	X	X	X	
Induction of labour									X	X	X	
Duration of labour									X	X	X	
**C- Medical risks related to previous pregnancies**												
Previous spontaneous abortions		X	X						X			
Previous stillbirths		X	X						X			
Previous neonatal deaths		X	X								X	
**D- Environmental and behavioural risks**												
Income domain of the SIMD	X	X	X	X	X	X	X	X	X	X	X	X
Urban/rural status of the area of residence	X	X	X	X	X	X	X	X	X	X	X	X
Booking status (gestational age at booking, booking before 25 weeks)	X			X	X	X	X	X	X	X	X	X

For peer review only

For peer review only - http://bmjopen.bmj.com/site/about/guidelines.xhtml

Smoking status (Before and during pregnancy)	X	X	X	X	X	X	X	X	X	X	X	
Typical weekly alcohol consumption (Before and during pregnancy)	X	X	X	X	X	X	X	X	X	X	X	X
Drug misuse during this pregnancy	X	X	X	X	X	X	X	X	X	X	X	X
**II- Unmeasured covariates**												
Maternal weight gain												
Maternal nutrition												
Maternal education												
Maternal exposure to stress												
Maternal physical activity												
Exposure to toxic substances												
Birth interval												
History of pre-term birth												
**Statistical methods**												
Multilevel linear regression	X	X		X			X					
Multilevel binomial logistic regression			X			X			X		X	X
Multilevel multinomial logistic regression					X			X		X		

For peer review only

X: variables which will be considered for adjustment in the statistical analysis

For peer review only

For peer review only - http://bmjopen.bmj.com/site/about/guidelines.xhtml

Economic analysis

The cost-effectiveness analysis will be based around relating the estimated cost of the intervention (£190 for HiP grant plus the costs of administering the grant) to the observed benefits of the programme (birthweight changes and changes in secondary endpoints such as stillbirths) from the natural experiment. As part of the project an economic model will be developed based on a review of the literature to relate birthweight changes (and any secondary outcomes affected by the HiP grant identified in this study) to long-term cost and health outcomes (in terms of QALYs). Other potential outcomes (such as the effect of birthweight on long term educational outcomes) will be summarised, but may not be included in the cost-per-QALY analysis. The review will inform only the relationship between birthweight and long-term outcomes, the effectiveness of the HiP grant will be taken only from the current study.

The perspective taken will be that of the UK National Health Service in the first instance. For this particular intervention it will be important to consider two further perspectives: the broader Public Sector (due to the relationship between LBW and social care/educational development), and society as whole (since the HiP grant is a transfer payment and therefore there is no net cost to society of transferring the grant from Government to individuals beyond the administration costs).

Of particular interest will be the relative cost-effectiveness of the programme between different socioeconomic groups identified in the main analysis. This may lead to differential policy recommendations for different socioeconomics groups. Uncertainty in the modelling of long-term outcomes will be subject to extensive sensitivity analysis to explore the robustness of the cost-effectiveness analysis.

For peer review only

For peer review only - http://bmjopen.bmj.com/site/about/guidelines.xhtml

IMPLICATIONS

Maternal nutrition plays a crucial role in influencing fetal growth and birth outcomes. It is a modifiable risk factor of public health importance in the effort to prevent adverse birth outcomes, particularly among low-income populations.[24] According to Barker, "*the seeds of inequalities in health in the next century are being sown today, in inner cities and other communities where adverse influences impact upon the growth, nutrition and health of mothers and their infants*".[71]

The HiP grant was cash given to the pregnant women with no constraint on its use. However, whether cash transfers are more efficient than "vouchers" or subsidies, which try to target the "appropriate expenditure", remains a controversial topic in economics.[72] This is because vouchers, for example, free up disposable income if they displace planned expenditure. This evaluation study may show the HiP grant increased birthweight across the population. If so, then a benefit would be to recommend the re-introduction of a universal cash transfer or, if we believed more evidence was needed that the HiP grants were delivering this benefit, the development of a randomized controlled trial for a similar cash transfer. An additional benefit will be the relative cost-effectiveness of the HiP grant between different socioeconomic groups identified in the main analysis. This may lead to differential policy recommendations for different socioeconomic groups with consequent reduction in health inequalities.

For peer review only - http://bmjopen.bmj.com/site/about/guidelines.xhtml

ETHICS AND DISSEMINATION

Ethical approval is not required as there is no primary data collection. Indeed, the information from maternal and birth records from all hospitals in Scotland are routinely collected. Approval for data collection, storage and release for research purpose has been given (6th May 2014; PAC38A/13) by the Privacy Advisory Committee, an Advisory Committee to NHS National Service Scotland and the Registrar General.

The results of this study will be disseminated through peer-reviewed publications in public health research journals, national and international conferences.

For peer review only

For peer review only - http://bmjopen.bmj.com/site/about/guidelines.xhtml

AUTHOR'S CONTRIBUTIONS

Ruth Dundas was involved in the conception of the study design, literature search and prepared the first draft of the study protocol. She will contribute to the statistical analysis and the interpretation of the results.

Samiratou Ouédraogo contributed to the literature search and the preparation of the present protocol paper. She will be responsible for the statistical analysis and contribute to the interpretation of the results.

Lyndal Bond was involved in the conception of the study design. She will contribute to the interpretation of the results.

Andrew H Briggs designed the health economic analysis aspect of the protocol. He will oversee the cost-effectiveness analysis and assist with writing the health economic paper.

James Chalmers was involved in the conception of the study design.

Ron Gray was involved in the conception of the study design. He will contribute to the interpretation of the results.

Rachael Wood will contribute to the interpretation of the results.

Alastair H Leyland was involved in the conception of the study design, literature search and contributed to all sections. He will contribute to the statistical analysis and the interpretation of the results.

All authors read and approved the final manuscript.

For peer review only

For peer review only - http://bmjopen.bmj.com/site/about/guidelines.xhtml

COMPETING INTERESTS

We declare that we have no conflicts of interest.

For peer review only

For peer review only - http://bmjopen.bmj.com/site/about/guidelines.xhtml

FUNDING STATEMENT

This project was funded by the National Institute for Health Research Public Health Research Board (project number 12/3070/02). The Social and Public Health Sciences Unit is core funded by the Medical Research Council (MC_UU_12017/5) and the Chief Scientist Office of the Scottish Government Health Directorates (SPHSU2).

The views and opinions expressed therein are those of the authors and do not necessarily reflect those of the Public Health Research Board, NIHR, NHS or the Department of Health.

For peer review only

REFERENCE LIST

1. Frankel S, Elwood P, Sweetnam P, et al. Birthweight, body-mass index in middle age, and incident coronary heart disease. Lancet 1996;348(9040):1478-80.

2. Leon DA, Lithell HO, Vagero D, et al. Reduced fetal growth rate and increased risk of death from ischaemic heart disease: cohort study of 15 000 Swedish men and women born 1915-29. BMJ 1998;317(7153):241-5.

3. Reyes L, Manalich R. Long-term consequences of low birth weight. Kidney Int Suppl 2005(97):S107-11.

4. Class QA, Rickert ME, Lichtenstein P, et al. Birth weight, physical morbidity, and mortality: a population-based sibling-comparison study. Am J Epidemiol 2014;179(5):550-8.

5. Hendryx M, Luo J, Knox SS, et al. Identifying multiple risks of low birth weight using person-centered modeling. Womens Health Issues 2014;24(2):e251-6.

6. Barker DJ. Birth weight and hypertension. Hypertension 2006;48(3):357-8.

7. Barker DJ, Eriksson JG, Forsen T, et al. Fetal origins of adult disease: strength of effects and biological basis. Int J Epidemiol 2002;31(6):1235-9.

8. Barker DJ. In utero programming of chronic disease. Clin Sci (Lond) 1998;95(2):115-28.

9. Barker DJ. The fetal and infant origins of adult disease. BMJ 1990;301(6761):1111.

10. Negrato C, Gomes M. Low birth weight: causes and consequences. Diabetol Metab Syndr 2013;5(1):49.

11. Valero De Bernabe J, Soriano T, Albaladejo R, et al. Risk factors for low birth weight: a review. Eur J Obstet Gynecol Reprod Biol 2004;116(1):3-15.

12. EURO-PERISTAT. “Babies’ health: mortality and morbidity during pregnancy and in the first year of life” in “The European Perinatal Health Report 2010”. 2013.

For peer review only - http://bmjopen.bmj.com/site/about/guidelines.xhtml

http://www.europeristat.com/images/doc/Peristat%202013%20V2.pdf. (Accessed 28 August 2014).

13. World Health Organisation. International Staticatical Classification of Diseases and Related Health Problems- ICD-10. 10th Revision. Geneva: World Health Organisation, 2010. http://www.who.int/classifications/icd/en/. (Accessed 28 August 2014).

14. OECD. "Infant health: Low birth weight", in Health at a Glance: Europe 2012. In: Publishing O, ed., 2012. http://www.oecd-ilibrary.org/docserver/download/8112121e.pdf?expires=1404136197&id=id&accname=guest&checksum=368A07F542E03A0442EFDEB9B002B6AE . (Accessed 28 August 2014).

15. Macfalane A, Dattani N. European Perinatal Health Report: highlights from a United Kingdom perspective. London, 2013. http://www.europeristat.com/images/Euro-peristat%20UK%20briefing.pdf. (Accessed 28 August 2014).

16. Madden D. The relationship between low birth weight and socioeconomic status in Ireland. J Biosoc Sci 2014;46(2):248-65.

17. Frimmel W, Pruckner GJ. Birth weight and family status revisited: evidence from Austrian register data. Health Econ 2014;23(4):426-45.

18. Huxley R. Smoking, birthweight, and mortality across generations. Eur Heart J 2013;34(44):3398-9.

19. Fairley L, Leyland AH. Social class inequalities in perinatal outcomes: Scotland 1980-2000. J Epidemiol Community Health 2006;60(1):31-6.

20. Weightman AL, Morgan HE, Shepherd MA, et al. Social inequality and infant health in the UK: systematic review and meta-analyses. BMJ Open 2012;2(3):e000964.

21. Juarez SP, Merlo J. Revisiting the effect of maternal smoking during pregnancy on offspring birthweight: a quasi-experimental sibling analysis in Sweden. PLoS One 2013;8(4):e61734.

For peer review only

22. Zdravkovic T, Genbacev O, McMaster MT, et al. The adverse effects of maternal smoking on the human placenta: a review. Placenta 2005;26(Suppl A):S81-6.

23. Villar J, Merialdi M, Gulmezoglu AM, et al. Nutritional interventions during pregnancy for the prevention or treatment of maternal morbidity and preterm delivery: an overview of randomized controlled trials. J Nutr 2003;133(5 Suppl 2):1606S-25S.

24. Abu-Saad K, Fraser D. Maternal nutrition and birth outcomes. Epidemiol Rev 2010;32(1):5-25.

25. Wu G, Imhoff-Kunsch B, Girard AW. Biological mechanisms for nutritional regulation of maternal health and fetal development. Paediatr Perinat Epidemiol 2012;26(Suppl 1):4-26.

26. Mason JB, Saldanha LS, Ramakrishnan U, et al. Opportunities for improving maternal nutrition and birth outcomes: synthesis of country experiences. Food Nutr Bull 2012;33(Suppl 2):S104-37.

27. Thangaratinam S, Rogozinska E, Jolly K, et al. Effects of interventions in pregnancy on maternal weight and obstetric outcomes: meta-analysis of randomised evidence. BMJ 2012;344:e2088.

28. Glassman A, Duran D, Fleisher L, et al. Impact of Conditional Cash Transfers on Maternal and Newborn Health. J Health Popul Nutr 2013;4:19.

29. Sutherland K, Christianson JB, Leatherman S. Impact of targeted financial incentives on personal health behavior: a review of the literature. Med Care Res Rev 2008;65(Suppl 6):36S-78S.

30. Marteau TM, Ashcroft RE, Oliver A. Using financial incentives to achieve healthy behaviour. BMJ 2009;338:b1415.

31. Giles EL, Robalino S, McColl E, et al. The effectiveness of financial incentives for health behaviour change: systematic review and meta-analysis. PLoS One 2014;9(3):e90347.

For peer review only - http://bmjopen.bmj.com/site/about/guidelines.xhtml

32. Brownell M, Chartier M, Au W, et al. Evaluation of the Healthy Baby Program. Winnipeg, MB: Manitoba Centre for Health Policy. 2010. http://mchp-appserv.cpe.umanitoba.ca/reference/Healthy_Baby.pdf. (Accessed 22 September 2014).

33. Neil G. Universal to selective. Transformation of the Welfare State: The Silent Surrender of Public Responsibility: Oxford University Press 2002:20.

34. Mkandawire T. Targeting and Universalism in poverty reduction. Secondary Targeting and Universalism in poverty reduction. 2005. http://www.unrisd.org/80256B3C005BCCF9/%28httpAuxPages%29/955FB8A594EEA0B0C12570FF00493EAA/$file/mkandatarget.pdf. (Accessed 28 August 2014).

35. Craig P, Cooper C, Gunnell D, et al. Using natural experiments to evaluate population health interventions: new Medical Research Council guidance. J Epidemiol Community Health 2012;66(12):1182-6.

36. Medical Research Council. Using natural experiments to evaluate population health interventions: guidance for producers and users of evidence. http://www.behaviourworksaustralia.org/wp-content/uploads/2012/10/NaturalExperimentsGuidance_MRC-guidance.pdf. (Accessed 28 August 2014).

37. Einarsdottir K, Kemp A, Haggar FA, et al. Increase in caesarean deliveries after the Australian Private Health Insurance Incentive policy reforms. PLoS One 2012;7(7):e41436.

38. Ramsay CR, Matowe L, Grilli R, et al. Interrupted time series designs in health technology assessment: lessons from two systematic reviews of behavior change strategies. Int J Technol Assess Health Care 2003;19(4):613-23.

39. Penfold RB, Zhang F. Use of interrupted time series analysis in evaluating health care quality improvements. Acad Pediatr 2013;13(6 Suppl):S38-44.

For peer review only - http://bmjopen.bmj.com/site/about/guidelines.xhtml

40. Administrative Data Liaison Service. Maternity and Neonatal Linked Database. http://www.adls.ac.uk/nhs-scotland/maternity-and-neonatal-linked-database/?detail. (Accessed 16 September 2014).

41. Kendrick SW and Clarke JA. The Scottish Record Linkage System. Health Bulletin 1993 51(2):72-79.

42. Administrative Data Liaison Service. Scottish Birth Record. http://www.adls.ac.uk/nhs-scotland/sbr-scottish-birth-record/?detail. (Accessed 16 September 2014).

43. Administrative Data Liaison Service. Birth registrations. http://www.adls.ac.uk/national-records-of-scotland/birth-registrations/?detail. (Accessed 16 September 2014).

44. Information Service Division. Birth in Scottish Hospitals. http://www.isdscotland.org/Health-Topics/Maternity-and-Births/Births/Background.asp. (Accessed 16 September 2014).

45. General Register Office for Scotland. Vital Events-Births. http://www.gro-scotland.gov.uk/statistics/theme/vital-events/births/index.html. (Accessed 16 September 2014).

46. National Health Service Scotland. Data Quality Assurance Assessment of Maternity Data (SMR02). Scotland report April 2010. http://www.isdscotlandarchive.scot.nhs.uk/isd/files//DQA-Assessment-of-Maternity-Data-SMR02-2008-to-2009.pdf. (Accessed 11 September 2014).

47. Taulbut M, Walsh D. Poverty, parenting and poor early years' experiences in Scotland, England and three city regions. 2013. http://socialwelfare.bl.uk/subject-areas/services-client-groups/families/glasgowcentreforpopulationhealth/164762Poverty__parenting_and_poor_health.pdf. (Accessed 11 September 2014).

48. Carstairs V, Morris R. Deprivation: explaining differences in mortality between Scotland and England and Wales. Brit Med J 1989;299:886–889.

For peer review only - http://bmjopen.bmj.com/site/about/guidelines.xhtml

49. NHS National Services Scotland. Births in Scottish Hospital: Smoking and Pregnancy http://www.isdscotlandarchive.scot.nhs.uk/isd/2911.html. (Accessed 16 September 2014).

50. General Register Office for Scotland. Vital Events-General Background Informations. http://www.gro-scotland.gov.uk/statistics/theme/vital-events/births/index.html. (Accessed 16 September 2014).

51. General Register Office for Scotland. Vital Events-General Background Informations: Occupation and social class / socio-economic classification. http://www.gro-scotland.gov.uk/files2/stats/vital-events/ve-occupation-social-classification.pdf. (Accessed 16 September 2014).

52. The National Statistics Socio-economic Classification (NS-SEC rebased on the SOC2010). http://www.ons.gov.uk/ons/guide-method/classifications/current-standard-classifications/soc2010/soc2010-volume-3-ns-sec--rebased-on-soc2010--user-manual/index.html. (Accessed 28 August 2014).

53. Aspinall PJ, Jacobson B. Why poor quality of ethnicity data should not preclude its use for identifying disparities in health and healthcare. Qual Saf Health Care 2007;16(3):176-80.

54. Scholmerich VL, Erdem O, Borsboom G, et al. The association of neighborhood social capital and ethnic (minority) density with pregnancy outcomes in the Netherlands. PLoS One 2014;9(5):e95873.

55. Margerison-Zilko C. The Contribution of Maternal Birth Cohort to Term Small for Gestational Age in the United States 1989-2010: an Age, Period, and Cohort Analysis. Paediatr Perinat Epidemiol 2014;28(4):312-21.

56. The Scottish Government. Scottish Index of Multiple Deprivation 2012: A national Statistics publication for Scotland 2012. http://22fa0f74501b902c9f11-8b3fbddfa1e1fab453a8e75cb14f3396.r26.cf3.rackcdn.com/simd_448749_v7_20121217.pdf. (Accessed 28 August 2014).

For peer review only - http://bmjopen.bmj.com/site/about/guidelines.xhtml

57. The Scottish Government. Scottish Index of Multiple Deprivation 2012: Technical notes. http://simd.scotland.gov.uk/publication-2012/technical-notes/domains-and-indicators/income-domain/. (Accessed 16 September 2014).

58. Pattenden S, Dolk H, Vrijheid M. Inequalities in low birth weight: parental social class, area deprivation, and "lone mother" status. J Epidemiol Community Health 1999;53(6):355-8.

59. Spencer N, Bambang S, Logan S, et al. Socioeconomic status and birth weight: comparison of an area-based measure with the Registrar General's social class. J Epidemiol Community Health 1999;53(8):495-8.

60. Fairley L, Dundas R, Leyland AH. The influence of both individual and area based socioeconomic status on temporal trends in Caesarean sections in Scotland 1980-2000. BMC Public Health 2011;11:330.

61. Kent ST, McClure LA, Zaitchik BF, et al. Area-level risk factors for adverse birth outcomes: trends in urban and rural settings. BMC Pregnancy Childbirth 2013;13:129.

62. Shankardass K, O'Campo P, Dodds L, et al. Magnitude of income-related disparities in adverse perinatal outcomes. BMC Pregnancy Childbirth 2014;14:96.

63. Sterne JA, White IR, Carlin JB, et al. Multiple imputation for missing data in epidemiological and clinical research: potential and pitfalls. BMJ 2009;338:b2393.

64. Shadish WR, Cook TD, Campbell DT. Quasi-experiments: Interrupted time-series designs. In Experimental and quasi-experimental designs for generalized causal inference. Boston: Houghton-Mifflin 2002:171-206.

65. Rothman KJ. No adjustments are needed for multiple comparisons. Epidemiology 1990;1(1):43-6.

66. Greenland S. Multiple comparisons and association selection in general epidemiology. Int J Epidemiol 2008;37(3):430-4.

For peer review only

67. National Service Division. Pregnancy Screening Programmes. http://www.nsd.scot.nhs.uk/services/screening/pregscreening/index.html. (Accessed 28 August 2014)

68. NHS Health Scotland. Your guide to screening tests during pregnancy. http://www.healthscotland.com/uploads/documents/3985-YourGuideToScreeningTestsDuringPregnancy_1.pdf. (Accessed 28 August 2014).

69. The Scottish Government. NHS Performance Targets.

http://www.scotland.gov.uk/Topics/Health/Quality-Improvement-Performance/NHS-Performance-Targets. (Accessed 16 September 2014).

70. Mackay DF, Nelson SM, Haw SJ, et al. Impact of Scotland's smoke-free legislation on pregnancy complications: retrospective cohort study. PLoS Med 2012;9(3):e1001175.

71. Barker DJ. The foetal and infant origins of inequalities in health in Britain. J Public Health Med 1991;13(2):64-8.

72. Gentilini U. Cash and food transfers: A Primer. World Food Programme. 2007. https://www.wfp.org/sites/default/files/OP18_Cash_and_Food_Transfers_Eng%2007.pdf (Accessed 16 September 2014).

For peer review only

PROJECT PROTOCOL

Title: Evaluation of Health in Pregnancy grants in Scotland: a protocol for a natural experiment

Authors: Ruth Dundas[1], Samiratou Ouédraogo[1], Lyndal Bond[2], Andrew H Briggs[3], James Chalmers[4], Ron Gray[5], Rachael Wood[4], Alastair H Leyland[1]

Affiliations:

[1] MRC/CSO Social and Public Health Sciences Unit, University of Glasgow, Glasgow, UK

[2] Centre of Excellence in Intervention and Prevention Science, Melbourne, Australia

[3] Health Economics and Health Technology Assessment, University of Glasgow, Glasgow, UK

[4] Information Services Division, NHS National Services Scotland, Edinburgh, UK

[5] National Perinatal Epidemiology Unit, University of Oxford, UK

Correspondence to:

Ruth Dundas

MRC/CSO Social and Public Health Sciences Unit, University of Glasgow

200 Renfield Street, Glasgow G2 3QB, Scotland

Tel: +44 141 353 7541 Email: ruth.dundas@glasgow.ac.uk

Keywords: Low birthweight; interrupted time series; multilevel analysis; Health in Pregnancy grant; natural experiment.

For peer review only - http://bmjopen.bmj.com/site/about/guidelines.xhtml

ABSTRACT

Introduction

A substantial proportion of low birthweight (LBW) is attributable to the mother's cultural and socioeconomic circumstances. Early childhood programmes have been widely developed to improve child outcomes. In the UK, the Health in Pregnancy (HiP) grant, a universal conditional cash transfer of £190, was introduced for women reaching the 25th week of pregnancy with a due date on/or after 6th April 2009 and subsequently withdrawn for women reaching the 25th week of pregnancy on/or after 1st January 2011. The current study focuses on the evaluation of the effectiveness and cost-effectiveness of the HiP grant.

Methods and analysis

The population under study will be all singleton births in Scotland over the periods of January 2004 to March 2009 (pre-intervention), April 2009 to April 2011 (intervention) and May 2011 to December 2013 (post-intervention). Data will be extracted from the Scottish maternity and neonatal database. The analysis period 2004-2013 should yield over 585,000 births. The primary outcome will be birthweight among singleton births. Other secondary outcomes will include gestation at booking, booking before 25 weeks; measures of size and stage; gestational age at delivery; weight-for-dates, term at birth; birth outcomes and maternal smoking. The main statistical method we will use is interrupted time series. Outcomes will be measured on individual births nested within mothers, with mothers themselves clustered within datazones. Multilevel regression models will be used to determine whether the outcomes changed during the period in which the HiP grants was in effect. Subgroup analyses will be conducted for those groups most likely to benefit from the payments.

Ethics and dissemination

Approval for data collection, storage and release for research purpose has been given (6th of May 2014, PAC38A/13) by the Privacy Advisory Committee. The results of this study will be disseminated through peer-reviewed publications in journals, national and international conferences.

For peer review only - http://bmjopen.bmj.com/site/about/guidelines.xhtml

ARTICLE SUMMARY

Article focus

- To evaluate the effectiveness and cost-effectiveness of Health in Pregnancy grant (HiP) in Scotland.

Key messages

- We will assess the difference in birthweight for babies born to those mothers who were eligible for the HiP grant with babies born before the HiP grant was introduced or after it was withdrawn using interrupted time series multilevel analysis.

- Subgroup analyses will be conducted for those groups seen as having the greatest potential to benefit from the payments such as those living in the most deprived areas, those in the lowest social classes, lone mothers, primiparous women, teen mothers, mothers from ethnic minorities and selected combinations of these groups.

- As part of the project an economic model will be developed based on a review of the literature to relate birthweight changes to long-term cost and health outcomes (in terms of QALYs).

Strengths and limitations of this study

The strengths of this study are:

- This is the first study evaluating the effect and the cost-effectiveness of the HiP grant in Scotland. It will use routinely available vital event and maternal and neonatal health records that are known to have high completeness and accuracy.
- The evaluation of the HiP grant using an interrupted time series design will enable us to analyse birthweight trends in Scotland and detect whether the intervention has had an effect over and above the underlying temporal trend. The use of interrupted time series will overcome other biases such as the autocorrelation of repeated measurements (measurement taken close together are related), seasonal effects (birthweight varies according to month of birth), the duration of the intervention (we will have pre-intervention intervention and post-intervention), and random variation in the measurement (birthweight).

The limitations of this study

For peer review only

- This study will evaluate the effect and the cost-effectiveness of the HiP grant, birthweight and other birth outcomes in Scotland using obstetric as well as maternal data. However, these outcomes may be influenced by many factors, not all of which are routinely captured (such as maternal diet, maternal work and psychological stress, abuse, exposure to toxic substance).[11]
- The HiP grant was money given to pregnant women with no constraint on its use. The use of routine data gives us no indication on how the money was spent.

For peer review only

For peer review only - http://bmjopen.bmj.com/site/about/guidelines.xhtml

INTRODUCTION

Low birthweight (LBW) due to preterm birth, intrauterine growth restriction, or being born small for gestational age (SGA), is commonly associated with perinatal mortality and impaired development[1-11]. Despite the improvement of the mortality in LBW babies over the past three decades, more than 70% of all neonatal mortality in Europe is found in infants weighing less than 2500 grams (g).[12]

LBW refers to birth weights below 2500 g,[13] irrespective of the gestational age of the infant. In most developed countries, the prevalence of LBW has increased due to several reasons: the number of multiple births with the increased risks of pre-term births and low birth weight partly as a result of the rise in fertility treatments; older age at childbearing and increases in the use of delivery management techniques such as induction of labour and caesarean delivery, which have increased the survival rates of low birth weight babies.[14] About one in 20 babies born in Europe in 2010 weighed less than 2500 g at birth.[12] With LBW estimated at 7.0% of live births in England and Wales, 6.5% in Scotland and 5.7% of live births in Northern Ireland, prevalence of LBW in the countries of the United Kingdom (UK) tended to be higher than in the rest of Europe.[12-15] Moreover, compared with other Western European countries, the UK has an incidence of LBW (<2500g) and very LBW (<1500g) in the top third. The proportion of pre-term birth (<37 weeks) is also high compared with other Western European countries.

Considerable attention has been focused on the causal determinants of LBW, in order to identify potentially modifiable factors. A substantial proportion of LBW is attributable to the mother's cultural and socioeconomic circumstances such as socioeconomic status (SES), harmful behaviours (smoking and excessive alcohol consumption) and poor nutrition during pregnancy.[11 16-18] In a study of social class inequalities in perinatal outcomes in Scotland, Fairley and Leyland[19] reported a percentage of 5.8% LBW in unskilled social class (V) compared to 2.9% in professional social class (I) between 1995 and 2000. The systematic review and meta-analyses of social inequality and infant health in the UK performed by Weightman et al.[20] found that the odds ratio for low birth weight was 1.79 (95% CI 1.43 to 2.24) in the lowest compared to the highest social class. This effect may vary with maternal factors such as age and smoking status. Smoking during pregnancy reduces birthweight by 162 to 377 g, depending on daily consumption (larger reduction for heavy smokers) and the trimester in which exposure occurs (larger reduction during the last trimester).[18,21-22]

For peer review only - http://bmjopen.bmj.com/site/about/guidelines.xhtml

The association between maternal nutrition and birth outcome is complex and is influenced by many biologic, socioeconomic, and demographic factors, which vary widely in different populations.[23-24] However, it has been reported that favourable prenatal nutrition associated with adequate prenatal care can have a positive impact on birth outcomes and morbidity in adult life.[25-26] Indeed, the developmental model of the origins of chronic disease proposes the causal influence of undernutrition *in utero* on coronary heart disease and stroke in adult life.[7-9] An improvement in fetal nutrition may therefore have far reaching consequences in terms of the prevention of disease. A review of maternal nutrition and birth outcomes identified improving maternal nutrition as being beneficial to the prevention of adverse birth outcomes in lower social class groups.[24]

A number of early childhood programmes have been developed to improve child outcomes. There is mixed evidence that these programmes do provide such improvement. In a meta-analysis of the effect of interventions in pregnancy on maternal and obstetric outcomes, Thangaratinam et al. concluded that dietary and lifestyle interventions in pregnancy reduced maternal gestational weight gain but had less effect on outcomes related to fetal weight and other morbidity and mortality.[27] Glassman et al.[28] who reported the results of conditional cash transfer programmes increasingly being adopted and scaled in developing countries, found that the programmes have increased the uptake of maternal and newborn health services, especially skilled attendance at delivery and antenatal monitoring. However, the impact of the programs on maternal and newborn mortality has not been well documented. Therefore, they recommended more rigorous impact evaluations that document impact pathways and take factors, such as cost-effectiveness, into account. Other studies evaluating payments to influence health behaviour found financial incentives were effective in increasing infrequent behaviours such as attending clinic appointments particularly in low-income groups, and recommended payments as being more effective than information and less restrictive than legislation.[29-31] In Canada, Brownell et al. have evaluated a complex programme on a prenatal benefit provided to families on low income during pregnancy.[32] They found that the receipt of this prenatal benefit was associated with a reduction in incidence of both low birthweight babies and preterm births. They suggested that efforts should be made to ensure all low-income women receive the income supplement.

In the UK, the Health in Pregnancy (HiP) grant was introduced for women reaching the 25th week of pregnancy with a due date on/or after 6th April 2009. It was subsequently withdrawn for women reaching the 25th week of pregnancy on/or after 1st January 2011. The HiP grant

For peer review only - http://bmjopen.bmj.com/site/about/guidelines.xhtml

was a universal conditional cash transfer of £190 for women reaching 25 weeks of pregnancy if they had sought health advice from a doctor or midwife. It was designed to provide additional financial support, in the last months of pregnancy, towards a healthy lifestyle including diet, and it was suggested that the link to the requirement for pregnant women to seek health advice from a professional may provide a greater incentive for expectant mothers to seek the recommended health advice at the appropriate time. The grant was paid and administered by Her Majesty's Revenue and Customs (HMRC) on receipt of a claim form partly completed by the midwife or doctor. Advice was offered as normal by doctors and midwives. Payment was made directly into a bank account with a telephone helpline available to provide support through the claims process including options for payment in the event of difficulties opening a bank account. Take up of the grant was said to be about the same level as for child benefit (98-99%, personal communication, HMRC).

The current study focuses on the evaluation of the effectiveness and cost-effectiveness of the HiP grant. As a primary outcome, we will consider the difference in birthweight for babies born to those mothers who were eligible for the HiP grant with babies born before the HiP grant was introduced or after it was withdrawn. Specific questions the research project will address are:

- Were there differential impacts of the intervention for particular subgroups defined by socioeconomic (defined in terms of both area deprivation and individual occupational social class), demographic (marital status, age, maternal height), or obstetric (parity, previous caesarean section) factors or for selected combination of these groups?
- Was the HiP grant cost-effective? How did cost-effectiveness vary across important subgroups identified as having differential outcomes?

The principle of universalism in the allocation of social benefits, that is the availability of social benefits to everyone as of right, is contrasted with allocation on a selective basis (targeted) in which benefits are allocated on the basis of need as determined by means testing of income.[33] The advantage of universal benefits is that they are easy to administer and can be efficiently delivered. The major disadvantage is that they are expensive, because they are delivered to those who do not need them as much as to those who do. However, the use of targeting involves some mechanism that discriminates between the poor and the non-poor. As such it always runs the danger of committing either type I errors which occur when someone

For peer review only

who deserves the benefits is denied them (underpayment, false positives), or type II errors, which occur when benefits are paid to someone who does not deserve them (overpayment, leakage).[34] The HiP grant represented an attempt to influence behaviour – appropriate and timely receipt of antenatal care advice – by means of a relatively modest, universally applied cash transfer. The evaluation of the effectiveness of such a payment may inform other policies aiming to change behaviour.

For peer review only

For peer review only - http://bmjopen.bmj.com/site/about/guidelines.xhtml

METHODS AND ANALYSIS

Study design

The HiP grant will be evaluated as a natural experiment using interrupted time series analysis to compare outcomes before the introduction of the intervention in Scotland and immediately after its withdrawal with those during the period for which it existed.

The Medical Research Council (MRC) has issued guidelines regarding the use of natural experiments to evaluate population health interventions when exposure to the intervention has not been manipulated by the researchers (Table 1).[35-36]

Table 1: Guidance for use of natural experiments to evaluate population health interventions

When to use a natural experimental approach?	How does the evaluation of HiP* grant meet these criteria?
▪ There is a reasonable expectation that the intervention will have a significant health impact, but scientific uncertainty about the size or nature of the effects.	▪ The HiP grant represented an attempt to influence behaviour – appropriate and timely receipt of antenatal care advice. With the sample size we are using in this evaluation study, we are able to detect small changes in birthweight.
▪ Natural experimental study is the most appropriate method for studying a given type of intervention.	▪ The HiP grant was a universally applied cash transfer available for all pregnant women with no discrimination between socioeconomic classes. This policy was not introduced using a randomised allocation
▪ It is possible to obtain the relevant data from an appropriate study population, comprising groups with different levels of exposure to the intervention.	▪ The uptake of the HiP grant was thought to be 98-99%. The linked Scottish birth dataset has 98% coverage of births and the primary outcome, birthweight, is well measured, 99.9% complete and accurate. Exposure is determined by the dates for which the HiP grant was in existence.
▪ The intervention or the principles behind it have the potential for replication, scalability or generalisability.	▪ The HiP grant is replicable everywhere in countries with similar health systems.

HiP* grant: Health in Pregnancy grant

For peer review only

The guidance advocates a number of designs including regression discontinuity designs such as interrupted time series. The interrupted time series approach is a powerful tool used for evaluating the impact of a policy change or quality improvement programme on the rate of an outcome in a defined population of individuals.[37-39] This approach will allow us to use a comparison group of pregnant women who delivered before the HiP grant was introduced, an intervention group who received the HiP grant and an additional post-intervention group who delivered after the HiP grant was withdrawn. It will also allow adjustment for seasonality, temporal trends and the socio-demographic and obstetric characteristics of the mother.

Study population

The population under study will be all singleton births in Scotland over the periods of January 2004 to March 2009 (pre-intervention), April 2009 to April 2011 (intervention) and May 2011 to December 2013 (post-intervention).

The Scottish maternity and neonatal database is a comprehensive record linkage system.[40-41] Probabilistic linkage procedures are used to add a unique identifier to all datasets to ensure all relevant records relating to an individual can be linked as required. It facilitates the linkage of a number of records from the system of Scottish Morbidity Records (SMR) including mother's obstetric records (SMR02) and the baby's birth and neonatal information from Scottish Birth Records (SBR).[42] Further links to the stillbirth and infant Death Survey and the National Records of Scotland (NRS) birth, stillbirth and infant death records can be carried out. The coverage is almost all births in Scotland.[43] From NRS data, we know of all registered births in Scotland (ie 100%). We have suitable record linked data on 98% of these births.[44] These will nearly all be hospital births; a fairly high proportion of the missing records are home births.

There is an average of about 56,000 births per year in Scotland.[45] The analysis period 2004-2013 should yield over 585,000 births. The analysis period 2006-2013 should yield over 455,000 births.

For peer review only - http://bmjopen.bmj.com/site/about/guidelines.xhtml

We have chosen to use data from the Scotland for this evaluation for the following reasons. First, data are available at a national level on the approximately 56,000 deliveries per year. Second, Scotland has a long history of collecting high quality routine data~~;~~. ~~the~~ The coverage, completeness and quality of the data are considered to be very high.[46] Third, the concentration of deprivation within parts of Scotland is unique within the UK.[47] ~~For~~ And ~~example six of the ten most deprived electoral constituencies in the UK are in Scotland and~~ using a UK wide Cartstairs index, the Scotland's population is over-represented in the bottom 5 deciles compared to England.[48] Fourth, data on smoking at booking have been routinely recorded in Scotland for a number of years.[49] This is not yet the case in England and Wales. The data can be linked to NRS civil registration data, which provides an estimate of completeness and contributes further information such as social class. The results will be generalisable to the rest of the UK and internationally. If the HiP grant proved beneficial in Scotland then there is every good reason to believe that a similar impact on outcomes could be achieved elsewhere and certainly in countries with similar health systems and comparable circumstances. Likewise, if the intervention was found to have been more effective for specific subgroups then we might expect subgroups to show greater benefits in other settings.

Study variables

Individual level and area level variables will be used in this study.

Individual level variables will include: birthweight, date of birth, sex, gestational age at delivery, preterm (delivery before the 37th week of pregnancy), weight-for-dates, 5 min Apgar score, crown to heel length, and head circumference. We will distinguish between spontaneous pre-term births and induced pre-term births. A potential reason for induced pre-term births is evidence of poor fetal growth; a proportion of these babies would become more severely growth retarded (more extremely small for gestational age) or stillborn.

Since maternal factors influence fetal growth,[11] individual level variables related to the mother will be examined: parity, age, height, weight at booking, diabetes, smoking status, gestation at booking, booking before 25 weeks, and marital status. Individual socioeconomic position will also be included using data from the birth registrations at NRS.[50] NRS collects occupation for both father and mother for births registered to married couples and jointly

For peer review only

For peer review only - http://bmjopen.bmj.com/site/about/guidelines.xhtml

registered by unmarried couples.[51] Only mother's occupation is recorded for sole registered births. The National Statistics Socio-economic Classification (NS-SEC)[40] [52] will be used to classify the individual socioeconomic position.

Marital status is an important variable as single mothers have consistently been shown to have poorer birth outcomes.[17-19] We are particularly interested in single mothers and social class. Social class for lone mothers is an amalgamation of socioeconomic position and lone parenthood.

Although the routinely collected data on ethnicity are incomplete and of dubious quality,[46,53] ethnicity remains important for birthweight and other neonatal outcomes.[54-55] We will therefore, within the constraints of the data, include ethnicity and undertake all analyses on the subgroup of mother from a minority ethnic background. Within this subgroup, we will examine the possibility of further distinction between ethnic groups.

Birthweight varies according to the socioeconomic status of the area of residence.[20] The Scottish Index of Multiple Deprivation (SIMD) will be used and included as area level variables in the analysis. The SIMD is the Scottish Government's official tool for identifying those places in Scotland suffering from deprivation.[43] [56] It is a weighted sum of different domains: income; employment; health; education; housing and geographical access (and crime, added in the SIMD 2012). The SIMD provides a comprehensive picture of material deprivation in small areas within Scotland. The index ranks 6505 areas from the most deprived to the least deprived and measures the degree of deprivation of an area relative to that of other areas. The areas employed by the SIMD are datazones and are small: the 6505 datazones have a mean population of 780 people. The reason for employing small area geography at this scale is to permit identification of relatively small pockets of deprivation. The health domain includes an indicator of the proportion of live singleton births that is LBW. The outcomes of this project include birthweight and LBW and so it would not be appropriate to use the health domain or the composite index which includes the health domain. The income domain will therefore be used to assess inequalities at the area level. This domain contributes 28% to the overall index and is highly correlated with the overall SIMD. The income domain of the SIMD identifies areas where there are concentrations of individuals and families living on low incomes. This is done by looking at the numbers of people, both adult and children, who are receiving, or are dependent on, benefits related to

For peer review only

income or tax credits.[57] Each mother will be assigned to a datazone and its income domain through her home postcode. Previous studies investigating inequalities in birthweight have shown that area deprivation performs as well as or better than individual social class in describing the extent of inequalities in the population.[58-59] However, Fairley et al. who studied the influence of both individual and area based socioeconomic status on temporal trends in Caesarean sections between 1980 and 2000 in Scotland found that maternal social class and area deprivation are different indicators of socioeconomic status which exhibit independent effects on the probability of a woman receiving a Caesarean section.[60] The multilevel analysis will allow us to analyse the effect of both parental social class and SMID and the effect of their interaction on birthweight.

We will adjust the analyses on the urban or rural status of the mother's area of residence. Indeed, Kent et al reported higher adverse birth outcome rates in isolated rural and more population dense areas.[61] They showed that these disparities are being maintained or increasing over time in Alabama. Shankardass et al also found that the patterns of association between socioeconomic position and LGA, spontaneous preterm birth and perinatal death varied depending on urbanicity in Nova Scotia (Canada).[62]

Outcome measures

Primary Outcome

The primary outcome will be birthweight among singleton births. This is influenced by many factors including maternal nutrition and one of the intentions of the HiP grant was to improve this.

Secondary Outcomes

The following secondary outcomes will be assessed:

- Gestation at booking,
- Booking before 25 weeks,
- Measures of size: crown to heel length, head circumference,
- Measures of stage: gestational age at delivery, weight-for-dates (standardised; small for gestational age babies are those weighted less than the 5th centile weight, or large

For peer review only - http://bmjopen.bmj.com/site/about/guidelines.xhtml

for gestational age weighted more than the 90th centile weight), term at birth (pre-term, babies are born at less than 37 weeks gestation; term babies are those born between 37-42 weeks gestation and post-term babies are born after 42 weeks gestation),

- Birth outcomes: mode of delivery, stillbirths, neonatal deaths, 5 minutes Apgar score. Although there is some debate concerning the robustness of the Apgar score as an outcome, it is in common use and we will therefore present results for this outcome within the context of the wide debate on the subject.

Due in part to the introduction of smoking ban in Scotland in 2006, an additional outcome of interest is maternal smoking. Maternal smoking is collected at booking and during pregnancy. The health advice given when receiving the HiP grant might have an impact on smoking rates during pregnancy over and above that of the smoke-free legislation. We will analyse the temporal change in smoking rate by socioeconomic class and its effect on outcomes.

Sample size

The data are clustered in small areas, 6505 datazones. The sample size calculation takes this clustering into account. Assuming an average of 56,000 singleton live births per year, and allowing for the clustering within the 6505 datazones in Scotland (average population 780 per datazone) with an estimated intraclass correlation coefficient of 0.05, we have power of 0.90 to detect an effect of 7g change in birthweight at a 95% significance level. This is not to say that 7g is a clinically important threshold; rather, it is indicative of the power of the study. The large national data set available to us will allow for subgroup analysis. In the 20% most deprived areas, we will have power of 0.80 to detect an effect of 13g; among the 26% of single mothers, we will have power of 0.80 to detect an effect of 11g. To put these small effects into context, 50g is the estimated mean birthweight reduction reported in the meta-analysis of the effect of interventions in pregnancy on maternal and obstetric outcomes.[27]

We anticipate item non-response for some outcomes and explanatory variables. Our primary outcome measure, birthweight, has a completion rate of 99.9%. There is high completion rate (<1.5% missing) for all obstetric variables,[46] with the exception of crown to heel length (15% missing) and head circumference (12% missing). The item non-response ranges from 8% for

For peer review only - http://bmjopen.bmj.com/site/about/guidelines.xhtml

maternal smoking to 20% for marital status. We will use multiple imputation to account for missing data and all analyses will compare the results of analyses of complete cases and multiple imputation.[63] Imputed models will be constructed such that they contain as many relevant predictor variables as possible. The more variables that are used the greater the amount of information available on which estimations are made. We will use all (or as many as possible) obstetric and maternal variables in an imputation model to predict the missing values. It is difficult to identify in advance the number of multiple imputed datasets we will need to construct but it is likely to be between 5 and 10. We will then analyse these datasets identically and combine the results to get the estimates and standard errors for the multiple imputed data. These results will be compared to the complete case analysis results.

It is difficult to be specific about the missing data mechanism until we see the data but much is likely to be missing completely at random (MCAR, e.g. certain hospitals are less likely to collect specific items) or missing at random (MAR, when the missingness is related to known variables and, conditional on these, is assumed to be unrelated to unmeasured variables).

Data analysis plan

Statistical analysis

Descriptive statistics of all variables will be presented as mean, standard deviation, minimum, median and maximum for continuous variables and as proportions when the variables are categorical.

The main statistical design we will use is interrupted time series.[64] Segmented regression analysis will allow an estimation of the size of the effect of the HiP grant at different time points, as well as changes in the trend of the effect over time after its implementation.

Outcomes will be measured on individual births, which are nested within mothers, with mothers themselves clustered within datazones and Health Boards. Multilevel univariable and multivariable models will be used to determine whether the outcomes changed during the period in which the HiP grants were in effect. Multilevel linear regression will be used when the outcome is continuous and multilevel binomial logistic regression when the outcome is dichotomous. Multilevel multinomial logistic regression will be used for the multi-category outcomes mode of delivery and 5 minutes Apgar score.

For peer review only

All analyses will be adjusted for temporal trends and seasonal variations in outcomes in addition to maternal age, sex of child, parity, marital status, height, weight at booking, diabetes, smoking status, gestation at booking, maternal diabetes, social class, maternal smoking and area deprivation.

The effect of HiP grant on birthweight might have a carryover effect after the withdrawal of the grant. In other words, post-intervention the slope in birthweight might not fall back to the same rate as pre-intervention. This could be due to women who have gave birth during the intervention subsequently having a birth postintervention but still heeding the health advice given during their first pregnancy. We will carry out an additional analysis only on primiparous women to avoid such contamination.

We will analyse pre-term births stratified by mode of delivery and stratified according to whether the birth was induced or spontaneous.

We will repeat the main analyses including (i.e adjusting for the effect of) ethnicity along with other covariates and compare the results with analyses excluding ethnicity to gauge the impact of this on our results. We note that the quality of this variable (including the completeness of recording) is poorer than for other variables and that only 1-2% of mothers delivering in Scotland are from minority ethnic backgrounds.

The simplest model for the intervention effect will include a dummy variable “intervention” covering the period from the introduction to the withdrawal of HiP grants, with adjustment for relevant factors such as marital status. (More complex models of the effect of the intervention will include an interaction of the intervention with the temporal trend). Before carrying out specific subgroup analysis, we will identify differential effects by fitting interaction terms. An assessment as to whether there is a differential effect of the intervention for single women, for example, will involve a test of the significance of the intervention between marital status and the intervention effect. If the interaction is significant this will aid our understanding of the generalisability to other populations, including the rest of the UK. Subgroup analyses will be conducted for those groups seen as having the greatest potential to benefit from the payments such as those living in the most deprived areas, those in the lowest social classes, lone mothers, primiparous women, teen mothers, mothers from ethnic minorities and selected combinations of these groups. For each group we will replicate the main analysis. This reduction in sample size for sub-group analyses will result in fewer

For peer review only - http://bmjopen.bmj.com/site/about/guidelines.xhtml

women/births being available but the same number of areas (datazones) will be analysed (apart from analyses restricted to those living in the most deprived areas).

An increase in birthweight, although desirable at a population level, may not be a beneficial outcome if the baby were already at risk of being large for gestational age (LGA). Separate subgroup analyses will therefore be conducted for women seen to be at high risk of delivering a LGA baby (women with diabetes) and for the remainder of the population. Given that some subgroups may contain small numbers, and bearing in mind the potential importance of the intervention, we will report the results of all subgroup analyses and not just those that reach statistical significance to avoid false negatives. The above process will involve conducting many tests, which will not be independent of each other. Rather than adjusting confidence intervals or p-values to account for this we will present the results of all analyses and caution the user regarding the interpretation of the results. Indeed, some statisticians recommend never correcting for multiple comparisons while analyzing data.[65-66] According to Rothman,[65] reducing the type I error for null associations increases the type II error for those associations that are not null. He recommends a policy of not making adjustments for multiple comparisons because it will lead to fewer errors of interpretation when the data under evaluation are not random numbers but actual observations on nature.

The HiP grant was introduced and withdrawn at the same time as other interventions that may have an impact on birthweight. Healthy Start is a means tested voucher scheme for pregnant women. If they are in receipt of certain benefits or under 18 years old, then they are eligible for free vitamins and vouchers to be spent on fruits and vegetables. This scheme replaced the means tested parts of the Welfare Food Scheme in the UK (including Scotland in 2006) and is still currently in place. During this period, there were policy changes in the optimal timing of first booking appointments due to changes in blood tests offered to pregnant women in the Pregnancy Screening Programme. These changes were first discussed in 2008 and had to be implemented by all Health Boards by March 2011.[67-68] Early booking is a HEAT target of the Scottish Government (Health improvement for the people of Scotland Efficiency and governance improvements, Access to services, Treatment appropriate to individuals H11.1).[69] At least 80% of pregnant women in each SIMD quintile will have booked for antenatal care by the 12th week of gestation by March 2015.

For peer review only - http://bmjopen.bmj.com/site/about/guidelines.xhtml

A further piece of legislation that may affect birthweight is the introduction of the smoking ban in public places in Scotland in March 2006. Mackay et al [70] reported a reduction in the prevalence of current smoking among women who conceived after the introduction of the legislation prohibiting smoking. They also reported a reduction in small and very small for gestational age, as well as in absolute low birth weight after the legislation. We will carry out a further analysis restricting the pre-intervention HiP grant period to April 2006-March 2009.

It is possible that harm may have occurred due to the cash transfer. The £190 was given to pregnant women with no restriction as to how it should be spent, and we do not know how the money was used. We are examining how the intervention group differed; birthweight could have reduced or increased. We will carry out 2-sided hypothesis tests to ensure that we are able to detect any such potential harmful effect.

We will conduct sensitivity analyses to increase the probability that any observed effect can be attributed to the HiP grant. The timing of the HiP grant is well defined and fixed, therefore using the interrupted time series approach any effects within that window can be observed. We plan to carry out 3 analyses for this.

1- We will extend this window for some months before April 2009 (births before the HiP grant was introduced).

2- We will extend this window for some months after April 2011 (births after the HiP grant was withdrawn).

3- We will extend the window both before and after the HiP grant period. In each case we would expect to see a dilution of any effects of the HiP grant.

The statistical analysis plan detailing the outcomes and the covariates, which will be considered for adjustment in the statistical models are presented in table 2.

For peer review only

For peer review only - http://bmjopen.bmj.com/site/about/guidelines.xhtml

Table 2: Analysis plan detailing the outcomes and the covariates that will be considered for adjustment in the statistical models

Covariates	Primary outcome	Secondary outcomes										
		Booking status		Measures of stage			Measures of size	Other birth outcomes				
	**Birth-weight**	Gestation at booking	Booking before 25 weeks	Gestational age at delivery	Term at birth	Weight-for-dates	Head circumference Crown to heel length	Mode of delivery	Still-birth	5 minutes Apgar score	Neonatal death	**Maternal smoking during this pregnancy**
**I- Measured covariates**												
**A- Socio-demographic determinants**												
**A-1. Related to the baby**												
Date of birth	X	X	X	X	X	X	X	X	X	X	X	X
Sex	X			X	X	X	X	X	X	X	X	
Gestational age at delivery	X						X	X	X	X	X	
Birthweight							X			X	X	
Mode of delivery										X	X	
**A-2. Related to the mother**												
Hip grant	X	X	X	X	X	X	X	X	X	X	X	X
Age	X	X	X	X	X	X	X	X	X	X	X	X
Weight at booking	X	X	X	X	X	X	X	X	X	X	X	
Height	X			X	X	X	X	X	X	X	X	
Ethnic group	X	X	X	X	X	X	X	X	X	X	X	X

For peer review only

Parity	X	X	X	X	X	X	X	X	X	X	X	X
Marital status	X	X	X	X	X	X	X	X	X	X	X	X
Social class	X	X	X	X	X	X	X	X	X	X	X	X
**B- Medical risks of the current pregnancy and before pregnancy**												
Diabetes	X	X	X	X	X	X	X	X	X	X	X	
Hypertension	X	X	X	X	X	X	X	X	X	X	X	
Infection	X			X	X	X	X	X	X	X	X	
Congenital anomalies	X			X	X	X	X	X	X	X	X	
Induction of labour									X	X	X	
Duration of labour									X	X	X	
**C- Medical risks related to previous pregnancies**												
Previous spontaneous abortions		X	X						X			
Previous stillbirths		X	X						X			
Previous neonatal deaths		X	X								X	
**D- Environmental and behavioural risks**												
Income domain of the SIMD	X	X	X	X	X	X	X	X	X	X	X	X
Urban/rural status of the area of residence	X	X	X	X	X	X	X	X	X	X	X	X
Booking status (gestational age at booking, booking before 25 weeks)	X			X	X	X	X	X	X	X	X	X

For peer review only

For peer review only - http://bmjopen.bmj.com/site/about/guidelines.xhtml

Smoking status (Before and during pregnancy)	X	X	X	X	X	X	X	X	X	X	X	
Typical weekly alcohol consumption (Before and during pregnancy)	X	X	X	X	X	X	X	X	X	X	X	X
Drug misuse during this pregnancy	X	X	X	X	X	X	X	X	X	X	X	X
**II- Unmeasured covariates**												
Maternal weight gain												
Maternal nutrition												
Maternal education												
Maternal exposure to stress												
Maternal physical activity												
Exposure to toxic substances												
Birth interval												
History of pre-term birth												
**Statistical methods**												
Multilevel linear regression	X	X		X			X					
Multilevel binomial logistic regression			X			X			X		X	X
Multilevel multinomial logistic regression					X			X		X		

For peer review only

X: variables which will be considered for adjustment in the statistical analysis

For peer review only

For peer review only - http://bmjopen.bmj.com/site/about/guidelines.xhtml

Economic analysis

The cost-effectiveness analysis will be based around relating the estimated cost of the intervention (£190 for HiP grant plus the costs of administering the grant) to the observed benefits of the programme (birthweight changes and changes in secondary endpoints such as stillbirths) from the natural experiment. As part of the project an economic model will be developed based on a review of the literature to relate birthweight changes (and any secondary outcomes affected by the HiP grant identified in this study) to long-term cost and health outcomes (in terms of QALYs). Other potential outcomes (such as the effect of birthweight on long term educational outcomes) will be summarised, but may not be included in the cost-per-QALY analysis. The review will inform only the relationship between birthweight and long-term outcomes, the effectiveness of the HiP grant will be taken only from the current study.

The perspective taken will be that of the UK National Health Service in the first instance. For this particular intervention it will be important to consider two further perspectives: the broader Public Sector (due to the relationship between LBW and social care/educational development), and society as whole (since the HiP grant is a transfer payment and therefore there is no net cost to society of transferring the grant from Government to individuals beyond the administration costs).

Of particular interest will be the relative cost-effectiveness of the programme between different socioeconomic groups identified in the main analysis. This may lead to differential policy recommendations for different socioeconomics groups. Uncertainty in the modelling of long-term outcomes will be subject to extensive sensitivity analysis to explore the robustness of the cost-effectiveness analysis.

For peer review only

IMPLICATIONS

Maternal nutrition plays a crucial role in influencing fetal growth and birth outcomes. It is a modifiable risk factor of public health importance in the effort to prevent adverse birth outcomes, particularly among low-income populations.[24] According to Barker, "*the seeds of inequalities in health in the next century are being sown today, in inner cities and other communities where adverse influences impact upon the growth, nutrition and health of mothers and their infants*".[71]

The HiP grant was cash given to the pregnant women with no constraint on its use. However, whether cash transfers are more efficient than "vouchers" or subsidies, which try to target the "appropriate expenditure", remains a controversial topic in economics.[72] However, economic theory would suggest that cash transfers are more efficient than "vouchers" or subsidies, which try to target the expenditure in the "appropriate expenditure". This is because vouchers, for example, free up disposable income if they displace planned expenditure. This evaluation study may show the HiP grant increased birthweight across the population. If so, then a benefit would be to recommend the re-introduction of a universal cash transfer or, if we believed more evidence was needed that the HiP grants were delivering this benefit, the development of a randomized controlled trial for a similar cash transfer. An additional benefit will be the relative cost-effectiveness of the HiP grant between different socioeconomic groups identified in the main analysis. This may lead to differential policy recommendations for different socioeconomic groups with consequent reduction in health inequalities.

For peer review only - http://bmjopen.bmj.com/site/about/guidelines.xhtml

ETHICS AND DISSEMINATION

Ethical approval is not required as there is no primary data collection. Indeed, the information from maternal and birth records from all hospitals in Scotland are routinely collected. Approval for data collection, storage and release for research purpose has been given (6th May 2014; PAC38A/13) by the Privacy Advisory Committee, an Advisory Committee to NHS National Service Scotland and the Registrar General.

The results of this study will be disseminated through peer-reviewed publications in public health research journals, national and international conferences.

For peer review only

For peer review only - http://bmjopen.bmj.com/site/about/guidelines.xhtml

AUTHOR'S CONTRIBUTIONS

Ruth Dundas was involved in the conception of the study design, literature search and prepared the first draft of the study protocol. She will contribute to the statistical analysis and the interpretation of the results.

Samiratou Ouédraogo contributed to the literature search and the preparation of the present protocol paper. She will be responsible for the statistical analysis and contribute to the interpretation of the results.

Lyndal Bond was involved in the conception of the study design. She will contribute to the interpretation of the results.

Andrew H Briggs designed the health economic analysis aspect of the protocol. He will oversee the cost-effectiveness analysis and assist with writing the health economic paper.

James Chalmers was involved in the conception of the study design.

Ron Gray was involved in the conception of the study design. He will contribute to the interpretation of the results.

Rachael Wood will contribute to the interpretation of the results.

Alastair H Leyland was involved in the conception of the study design, literature search and contributed to all sections. He will contribute to the statistical analysis and the interpretation of the results.

All authors read and approved the final manuscript.

For peer review only

COMPETING INTERESTS

We declare that we have no conflicts of interest.

For peer review only

For peer review only - http://bmjopen.bmj.com/site/about/guidelines.xhtml

FUNDING STATEMENT

This project was funded by the National Institute for Health Research Public Health Research Board (project number 12/3070/02). The Social and Public Health Sciences Unit is core funded by the Medical Research Council (MC_UU_12017/5) and the Chief Scientist Office of the Scottish Government Health Directorates (SPHSU2).

The views and opinions expressed therein are those of the authors and do not necessarily reflect those of the Public Health Research Board, NIHR, NHS or the Department of Health.

For peer review only

REFERENCE LIST

1. Frankel S, Elwood P, Sweetnam P, et al. Birthweight, body-mass index in middle age, and incident coronary heart disease. Lancet 1996;348(9040):1478-80.

2. Leon DA, Lithell HO, Vagero D, et al. Reduced fetal growth rate and increased risk of death from ischaemic heart disease: cohort study of 15 000 Swedish men and women born 1915-29. BMJ 1998;317(7153):241-5.

3. Reyes L, Manalich R. Long-term consequences of low birth weight. Kidney Int Suppl 2005(97):S107-11.

4. Class QA, Rickert ME, Lichtenstein P, et al. Birth weight, physical morbidity, and mortality: a population-based sibling-comparison study. Am J Epidemiol 2014;179(5):550-8.

5. Hendryx M, Luo J, Knox SS, et al. Identifying multiple risks of low birth weight using person-centered modeling. Womens Health Issues 2014;24(2):e251-6.

6. Barker DJ. Birth weight and hypertension. Hypertension 2006;48(3):357-8.

7. Barker DJ, Eriksson JG, Forsen T, et al. Fetal origins of adult disease: strength of effects and biological basis. Int J Epidemiol 2002;31(6):1235-9.

8. Barker DJ. In utero programming of chronic disease. Clin Sci (Lond) 1998;95(2):115-28.

9. Barker DJ. The fetal and infant origins of adult disease. BMJ 1990;301(6761):1111.

10. Negrato C, Gomes M. Low birth weight: causes and consequences. Diabetol Metab Syndr 2013;5(1):49.

11. Valero De Bernabe J, Soriano T, Albaladejo R, et al. Risk factors for low birth weight: a review. Eur J Obstet Gynecol Reprod Biol 2004;116(1):3-15.

12. EURO-PERISTAT. “Babies’ health: mortality and morbidity during pregnancy and in the first year of life” in “The European Perinatal Health Report 2010”. 2013.

For peer review only

http://www.europeristat.com/images/doc/Peristat%202013%20V2.pdf. (Accessed 28 August 2014).

13. World Health Organisation. International Staticatical Classification of Diseases and Related Health Problems- ICD-10. 10th Revision. Geneva: World Health Organisation, 2010. http://www.who.int/classifications/icd/en/. (Accessed 28 August 2014).

14. OECD. "Infant health: Low birth weight", in Health at a Glance: Europe 2012. In: Publishing O, ed., 2012. http://www.oecd-ilibrary.org/docserver/download/8112121e.pdf?expires=1404136197&id=id&accname=guest&checksum=368A07F542E03A0442EFDEB9B002B6AE . (Accessed 28 August 2014).

15. Macfalane A, Dattani N. European Perinatal Health Report: highlights from a United Kingdom perspective. London, 2013. http://www.europeristat.com/images/Euro-peristat%20UK%20briefing.pdf. (Accessed 28 August 2014).

16. Madden D. The relationship between low birth weight and socioeconomic status in Ireland. J Biosoc Sci 2014;46(2):248-65.

17. Frimmel W, Pruckner GJ. Birth weight and family status revisited: evidence from Austrian register data. Health Econ 2014;23(4):426-45.

18. Huxley R. Smoking, birthweight, and mortality across generations. Eur Heart J 2013;34(44):3398-9.

19. Fairley L, Leyland AH. Social class inequalities in perinatal outcomes: Scotland 1980-2000. J Epidemiol Community Health 2006;60(1):31-6.

20. Weightman AL, Morgan HE, Shepherd MA, et al. Social inequality and infant health in the UK: systematic review and meta-analyses. BMJ Open 2012;2(3):e000964.

21. Juarez SP, Merlo J. Revisiting the effect of maternal smoking during pregnancy on offspring birthweight: a quasi-experimental sibling analysis in Sweden. PLoS One 2013;8(4):e61734.

Field Code Changed

For peer review only

For peer review only - http://bmjopen.bmj.com/site/about/guidelines.xhtml

22. Zdravkovic T, Genbacev O, McMaster MT, et al. The adverse effects of maternal smoking on the human placenta: a review. Placenta 2005;26 (Suppl A):S81-6.

23. Villar J, Merialdi M, Gulmezoglu AM, et al. Nutritional interventions during pregnancy for the prevention or treatment of maternal morbidity and preterm delivery: an overview of randomized controlled trials. J Nutr 2003;133(5 Suppl 2):1606S-25S.

24. Abu-Saad K, Fraser D. Maternal nutrition and birth outcomes. Epidemiol Rev 2010;32(1):5-25.

25. Wu G, Imhoff-Kunsch B, Girard AW. Biological mechanisms for nutritional regulation of maternal health and fetal development. Paediatr Perinat Epidemiol 2012;26(-Suppl 1):4-26.

26. Mason JB, Saldanha LS, Ramakrishnan U, et al. Opportunities for improving maternal nutrition and birth outcomes: synthesis of country experiences. Food Nutr Bull 2012;33(2 Suppl 2):S104-37.

27. Thangaratinam S, Rogozinska E, Jolly K, et al. Effects of interventions in pregnancy on maternal weight and obstetric outcomes: meta-analysis of randomised evidence. BMJ 2012;344:e2088.

28. Glassman A, Duran D, Fleisher L, et al. Impact of Conditional Cash Transfers on Maternal and Newborn Health. J Health Popul Nutr 2013;4:19.

29. Sutherland K, Christianson JB, Leatherman S. Impact of targeted financial incentives on personal health behavior: a review of the literature. Med Care Res Rev 2008;65(6 Suppl 6):36S-78S.

30. Marteau TM, Ashcroft RE, Oliver A. Using financial incentives to achieve healthy behaviour. BMJ 2009;338:b1415.

31. Giles EL, Robalino S, McColl E, et al. The effectiveness of financial incentives for health behaviour change: systematic review and meta-analysis. PLoS One 2014;9(3):e90347.

For peer review only

For peer review only - http://bmjopen.bmj.com/site/about/guidelines.xhtml

32. Brownell M, Chartier M, Au W, et al. Evaluation of the Healthy Baby Program. Winnipeg, MB: Manitoba Centre for Health Policy. 2010. http://mchp-appserv.cpe.umanitoba.ca/reference/Healthy_Baby.pdf. (Accessed 22 September 2014).

Formatted: English (U.K.)

33. Neil G. Universal to selective. Transformation of the Welfare State: The Silent Surrender of Public Responsibility: Oxford University Press, 2002:20.

34. Mkandawire T. Targeting and Universalism in poverty reduction. . Secondary Targeting and Universalism in poverty reduction. 2005. http://www.unrisd.org/80256B3C005BCCF9/%28httpAuxPages%29/955FB8A594EEA0B0C12570FF00493EAA/$file/mkandatarget.pdf. (Accessed 28 August 2014).

Field Code Changed

35. Craig P, Cooper C, Gunnell D, et al. Using natural experiments to evaluate population health interventions: new Medical Research Council guidance. J Epidemiol Community Health 2012;66(12):1182-6.

36. Council MRMedical Research Council. Using natural experiments to evaluate population health interventions: guidance for producers and users of evidence. http://www.behaviourworksaustralia.org/wp-content/uploads/2012/10/NaturalExperimentsGuidance_MRC-guidance.pdf. (Accessed 28 August 2014).

Field Code Changed

37. Einarsdottir K, Kemp A, Haggar FA, et al. Increase in caesarean deliveries after the Australian Private Health Insurance Incentive policy reforms. PLoS One 2012;7(7):e41436.

38. Ramsay CR, Matowe L, Grilli R, et al. Interrupted time series designs in health technology assessment: lessons from two systematic reviews of behavior change strategies. Int J Technol Assess Health Care 2003;19(4):613-23.

39. Penfold RB, Zhang F. Use of interrupted time series analysis in evaluating health care quality improvements. Acad Pediatr 2013;13(6 Suppl):S38-44.

For peer review only - http://bmjopen.bmj.com/site/about/guidelines.xhtml

40. Administrative Data Liaison Service. Maternity and Neonatal Linked Database. http://www.adls.ac.uk/nhs-scotland/maternity-and-neonatal-linked-database/?detail. (Accessed 16 September 2014).

41. Kendrick SW and Clarke JA. The Scottish Record Linkage System. Health Bulletin 1993 51(2):72-79.

42. Administrative Data Liaison Service. Scottish Birth Record. http://www.adls.ac.uk/nhs-scotland/sbr-scottish-birth-record/?detail. (Accessed 16 September 2014).

43. Administrative Data Liaison Service. Birth registrations. http://www.adls.ac.uk/national-records-of-scotland/birth-registrations/?detail. (Accessed 16 September 2014).

44. Information Service Division. Birth in Scottish Hospitals. http://www.isdscotland.org/Health-Topics/Maternity-and-Births/Births/Background.asp. (Accessed 16 September 2014).

45. General Register Office for Scotland. Vital Events-Births. http://www.gro-scotland.gov.uk/statistics/theme/vital-events/births/index.html. (Accessed 16 September 2014).

46. National Health Service Scotland. Data Quality Assurance Assessment of Maternity Data (SMR02). Scotland report April 2010. http://www.isdscotlandarchive.scot.nhs.uk/isd/files//DQA-Assessment-of-Maternity-Data-SMR02-2008-to-2009.pdf. (Accessed 11 September 2014).

47. Taulbut M, Walsh D. Poverty, parenting and poor early years' experiences in Scotland, England and three city regions. 2013. http://socialwelfare.bl.uk/subject-areas/services-client-groups/families/glasgowcentreforpopulationhealth/164762Poverty__parenting_and_poor_health.pdf. (Accessed 11 September 2014).

48. Carstairs V, Morris R. Deprivation: explaining differences in mortality between Scotland and England and Wales. Brit Med J 1989;299:886–889.

Field Code Changed

Field Code Changed

Field Code Changed

Field Code Changed

For peer review only - http://bmjopen.bmj.com/site/about/guidelines.xhtml

49. NHS National Services Scotland. Births in Scottish Hospital: Smoking and Pregnancy http://www.isdscotlandarchive.scot.nhs.uk/isd/2911.html. (Accessed 16 September 2014).

50. General Register Office for Scotland. Vital Events-General Background Informations. http://www.gro-scotland.gov.uk/statistics/theme/vital-events/births/index.html. (Accessed 16 September 2014).

51. General Register Office for Scotland. Vital Events-General Background Informations: Occupation and social class / socio-economic classification. http://www.gro-scotland.gov.uk/files2/stats/vital-events/ve-occupation-social-classification.pdf. (Accessed 16 September 2014).

52. The National Statistics Socio-economic Classification (NS-SEC rebased on the SOC2010). http://www.ons.gov.uk/ons/guide-method/classifications/current-standard-classifications/soc2010/soc2010-volume-3-ns-sec--rebased-on-soc2010--user-manual/index.html. (Accessed 28 August 2014).

53. Aspinall PJ, Jacobson B. Why poor quality of ethnicity data should not preclude its use for identifying disparities in health and healthcare. Qual Saf Health Care 2007;16(3):176-80.

54. Scholmerich VL, Erdem O, Borsboom G, et al. The association of neighborhood social capital and ethnic (minority) density with pregnancy outcomes in the Netherlands. PLoS One 2014;9(5):e95873.

55. Margerison-Zilko C. The Contribution of Maternal Birth Cohort to Term Small for Gestational Age in the United States 1989-2010: an Age, Period, and Cohort Analysis. Paediatr Perinat Epidemiol 2014;28(4):312-21.

56. The Scottish Government. Scottish Index of Multiple Deprivation 2012: A national Statistics publication for Scotland 2012. http://22fa0f74501b902c9f11-8b3fbddfa1e1fab453a8e75cb14f3396.r26.cf3.rackcdn.com/simd_448749_v7_20121217.pdf. (Accessed 28 August 2014).

For peer review only

57. The Scottish Government. Scottish Index of Multiple Deprivation 2012: Technical notes. http://simd.scotland.gov.uk/publication-2012/technical-notes/domains-and-indicators/income-domain/. (Accessed 16 September 2014).

Field Code Changed

58. Pattenden S, Dolk H, Vrijheid M. Inequalities in low birth weight: parental social class, area deprivation, and "lone mother" status. J Epidemiol Community Health 1999;53(6):355-8.

59. Spencer N, Bambang S, Logan S, et al. Socioeconomic status and birth weight: comparison of an area-based measure with the Registrar General's social class. J Epidemiol Community Health 1999;53(8):495-8.

60. Fairley L, Dundas R, Leyland AH. The influence of both individual and area based socioeconomic status on temporal trends in Caesarean sections in Scotland 1980-2000. BMC Public Health 2011;11:330.

61. Kent ST, McClure LA, Zaitchik BF, et al. Area-level risk factors for adverse birth outcomes: trends in urban and rural settings. BMC Pregnancy Childbirth 2013;13:129.

62. Shankardass K, O'Campo P, Dodds L, et al. Magnitude of income-related disparities in adverse perinatal outcomes. BMC Pregnancy Childbirth 2014;14:96.

63. Sterne JA, White IR, Carlin JB, Spratt M, Royston P, Kenward MG, Wood AM, Carpenter JR. Multiple imputation for missing data in epidemiological and clinical research: potential and pitfalls. BMJ 2009;338:b2393.

64. Shadish WR, Cook TD, Campbell DT. Quasi-experiments: Interrupted time-series designs. In Experimental and quasi-experimental designs for generalized causal inference. Boston: Houghton-Mifflin 2002:171-206.

65. Rothman KJ. No adjustments are needed for multiple comparisons. Epidemiology 1990;1(1):43-6.

For peer review only

For peer review only - http://bmjopen.bmj.com/site/about/guidelines.xhtml

66. Greenland S. Multiple comparisons and association selection in general epidemiology. Int J Epidemiol 2008;37(3):430-4.

67. National Service Division. Pregnancy Screening Programmes. http://www.nsd.scot.nhs.uk/services/screening/pregscreening/index.html. (Accessed 28 August 2014)

68. NHS Health Scotland. Your guide to screening tests during pregnancy. http://www.healthscotland.com/uploads/documents/3985-YourGuideToScreeningTestsDuringPregnancy_1.pdf. (Accessed 28 August 2014).

69. The Scottish Government. NHS Performance Targets.

http://www.scotland.gov.uk/Topics/Health/Quality-Improvement-Performance/NHS-Performance-Targets. (Accessed 16 September 2014).

70. Mackay DF, Nelson SM, Haw SJ, et al. Impact of Scotland's smoke-free legislation on pregnancy complications: retrospective cohort study. PLoS Med 2012;9(3):e1001175.

71. Barker DJ. The foetal and infant origins of inequalities in health in Britain. J Public Health Med 1991;13(2):64-8.

72. Gentilini U. Cash and food transfers: A Primer. World Food Programme. 2007. https://www.wfp.org/sites/default/files/OP18_Cash_and_Food_Transfers_Eng%2007.pdf (Accessed 16 September 2014).

Field Code Changed

Field Code Changed

Field Code Changed

Field Code Changed

For peer review only

For peer review only - http://bmjopen.bmj.com/site/about/guidelines.xhtml
